# SpliceCOV: Supervised Filtering of RNA-Seq-Derived Transcript Features Improves Transcriptome Assembly

**DOI:** 10.3390/genes17101307

**Published:** 2026-10-20

**Authors:** Celine Hoh, Ida Shinder, Kuan-Hao Chao, Ales Varabyou, Mihaela Pertea

**Affiliations:** 1 Department of Computer Science, Johns Hopkins University, Baltimore, MD 21218, USA; 2 Center for Computational Biology, Johns Hopkins University, Baltimore, MD 21211, USA; 3 Cross Disciplinary Graduate Program in Biomedical Sciences, Johns Hopkins School of Medicine, Baltimore, MD 21205, USA; 4 Department of Biomedical Engineering, Johns Hopkins University, Baltimore, MD 21211, USA

**Keywords:** RNA-Seq, transcriptome assembly, splice sites, transcription boundaries, transcript annotation, machine learning, LightGBM

## Abstract

**Background/Objectives**: High-throughput RNA sequencing enables transcript discovery and quantification, but the resulting coverage profiles often contain substantial transcriptional noise, making it difficult to distinguish genuine transcript features from spurious signals. These noisy signals can propagate into transcriptome assemblies, increasing the number of false transcript models and reducing annotation precision. **Methods**: We present SpliceCOV, a supervised framework for filtering RNA-Seq-derived transcript features. Rather than performing general-purpose segmentation of RNA-Seq coverage, SpliceCOV uses coverage- and junction-derived features to distinguish likely genuine splice sites and transcript boundaries from noisy candidates. We benchmarked SpliceCOV against established changepoint methods and evaluated whether SpliceCOV-filtered features improve downstream transcriptome assembly with StringTie. **Results**: Across human GTEx tissue datasets, SpliceCOV substantially reduced likely false-positive splice sites while retaining most annotated sites. We included segmentation-based methods as baselines to assess whether generic coverage changepoints can support RNA-Seq transcript-feature filtering. Because these methods are unsupervised whereas SpliceCOV is trained using annotation-derived labels, the comparisons are intended to assess suitability for this specific filtering task rather than to provide a general ranking of the underlying algorithms. Under this evaluation framework, SpliceCOV retained more annotation-supported splice sites at comparable levels of filtering. For transcript start and end sites, SpliceCOV reduced the majority of false positives while retaining substantial overlap with reference annotations. When SpliceCOV-filtered splice sites and transcript boundaries were supplied to StringTie, transcript-level precision increased across the evaluated tissues while the number of reference-matching transcripts remained broadly similar. The model also showed transferability across tissues and chromosomes, and could be applied to mouse RNA-Seq without retraining, although downstream assembly performance was lower than in human datasets. **Conclusions**: SpliceCOV provides a supervised approach for filtering noisy RNA-Seq-derived transcript features before transcriptome assembly. By improving the quality of splice-site and transcript-boundary evidence supplied to downstream assembly, SpliceCOV can increase transcript-level precision while preserving transcript recovery.

## 1. Introduction

Advances in high-throughput RNA sequencing have transformed transcriptome profiling by enabling the discovery of novel genes and transcripts [1], the characterization of alternative splicing across tissues [2], and improved estimation of transcript abundance [3,4,5]. However, RNA-Seq often captures substantial transcriptional noise, including intergenic reads, spurious splice junctions, and incompletely spliced RNAs. This noise can account for up to one-third of all transcripts assembled from RNA-Seq and can lead to the erroneous identification of novel transcripts or splicing events that do not reflect genuine biological processes [6,7,8,9,10,11]. As a result, RNA-Seq-based transcript discovery faces a central trade-off, whereby permissive approaches preserve sensitivity but can introduce many false-positive features, while aggressive filtering improves precision but risks removing genuine low-abundance or tissue-specific transcriptional events.

Changepoint detection is the task of identifying positions where the statistical properties of sequential data change abruptly, and it is widely used for signal segmentation [12]. It has been widely used in genomics, including for DNA copy number analysis [13,14,15], and more recently, to analyses of long-read nanopore RNA-sequencing data [16]. Prominent algorithms include Binary Segmentation [17] and the Pruned Exact Linear Time (PELT) algorithm [18], which uses dynamic programming with pruning to optimize a penalized segmentation objective. More recent exact methods include Generalized Functional Pruning Optimal Partitioning (GFPOP) [19], which improves efficiency through functional pruning using graph-constrained changepoint models. Other approaches, such as DeCAFS [20], address related challenges such as penalty selection, local fluctuations, and autocorrelated noise. These methods provide principled and complementary approaches for detecting changes in genomic signals.

However, a change in RNA-Seq coverage is not, by itself, equivalent to a biologically meaningful splice site, transcription start site, or transcription end site. RNA-Seq coverage is sparse, overdispersed, and affected by sequencing depth, library preparation, alignment, and tissue-specific expression [21]. Therefore, transcript-feature detection requires not only sensitivity to local coverage patterns, but also the integration of RNA-Seq-specific evidence, such as junction support and local coverage context.

SpliceCOV is designed to address this filtering problem. Rather than introducing another general-purpose segmentation algorithm, SpliceCOV focuses on a different problem: given RNA-Seq-derived candidate transcriptional features, determine which candidates are likely to reflect genuine transcript structure and which are more likely to represent transcriptional noise or technical artifacts. Splice sites and transcript boundaries present different candidate-detection problems. For splice sites, RNA-Seq alignments already provide a sensitive candidate set through observed splice junctions. Each junction identifies a candidate donor and acceptor site, and the main challenge is to determine whether each observed site is sufficiently supported by local RNA-Seq evidence. In this setting, SpliceCOV directly applies a trained LightGBM [22] classifier to candidate splice sites derived from the observed junction set, using coverage- and junction-derived features to distinguish supported splice sites from likely noisy candidates.

Transcript start and end sites (TSS/TES) require a different strategy because RNA-Seq alignments do not provide an analogous junction-derived candidate set for TSS/TES. For these features, SpliceCOV first uses a coverage-based heuristic to propose candidate transcript boundaries from directional changes in RNA-Seq coverage. These candidate boundaries are then passed to a separate LightGBM classifier trained on local coverage- and junction-derived features. In this part of the workflow, coverage transitions consistent with changepoints are useful for generating candidate TSS/TES positions, but the final objective remains supervised filtering rather than exhaustive segmentation of the RNA-Seq coverage signal.

In this study, we use these segmentation methods as task-relevant baselines to evaluate how well segmentation-derived changepoints can support RNA-Seq transcriptional feature filtering. We compare SpliceCOV with PELT, Segmentor3IsBack, and GFPOP for splice-site filtering, and with PELT, Segmentor3IsBack, and DeCAFS for TSS/TES detection. These comparisons assess how well segmentation-derived changepoints recover annotation-supported RNA-Seq features relative to SpliceCOV’s supervised filtering strategy, rather than evaluating the overall utility of these methods for their original purposes.

We benchmark SpliceCOV on bulk RNA-Seq data from the GTEx project [23] and evaluate its ability to recover annotated splice sites and transcript boundaries while reducing likely false positives, indicating that prior segmentation is not necessary and can limit sensitivity. We further test whether SpliceCOV-filtered features improve downstream transcriptome assembly when supplied to StringTie v.3.0.1 [24]. Across tissues, SpliceCOV increases precision without compromising transcript recovery, and the benefits are larger in tissues with greater sample depth, where aggregated RNA-Seq evidence provides stronger support for reproducible transcriptional features. We also evaluate generalization across chromosomes, tissues, and species, showing that a model trained on human RNA-Seq can improve splice-site filtering and transcriptome assembly in mice without retraining. Together, these results support SpliceCOV as a scalable supervised filtering approach for removing transcriptional noise from RNA-Seq-derived transcript features while preserving sensitivity for transcript discovery. SpliceCOV is open-source and available at https://github.com/celinehohzm/SpliceCOV (accessed on 10 October 2026).

## 2. Materials and Methods

### 2.1. SpliceCOV Framework and Implementation

**Figure 1:**
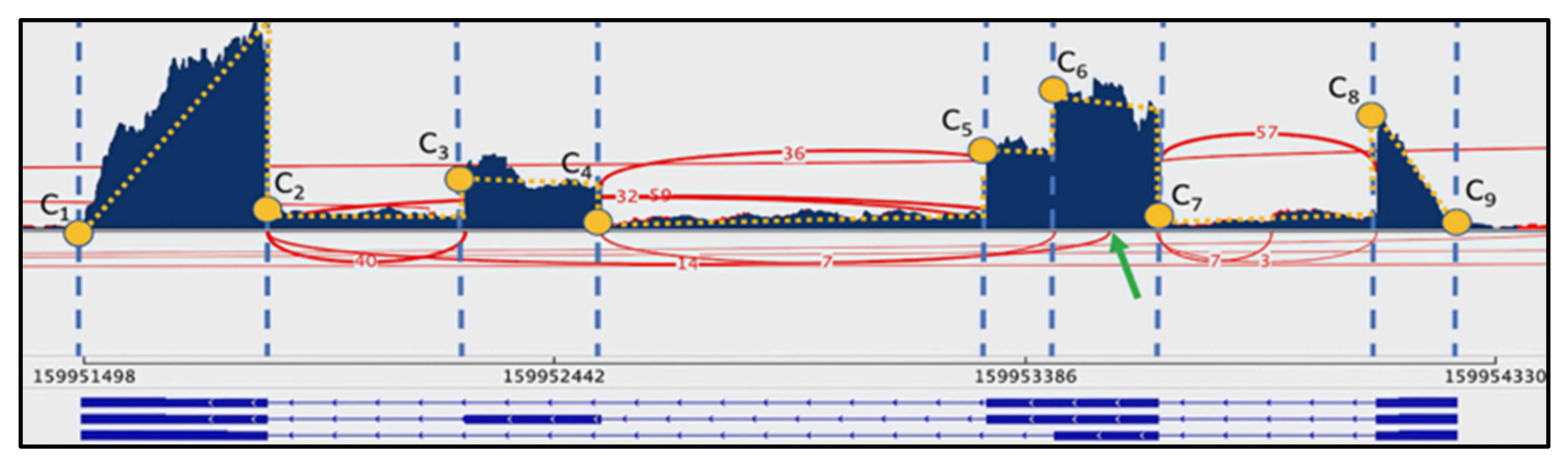
Representative changepoints (yellow) in the SLAMF9 gene’s RNA-Seq coverage, corresponding to transcriptional start/end sites (e.g., c1, c9) or splice sites (c2–c8). Red arcs represent observed splice junctions connecting donor and acceptor sites, with the numbers indicating the number of supporting junction reads. The green arrow indicates a spliced read that does not produce a changepoint, and it likely represents splicing noise. Three CHESS-annotated SLAMF9 transcripts are shown at the bottom in blue.

SpliceCOV is a supervised framework for filtering RNA-Seq-derived transcriptional features. Its goal is to distinguish candidate splice sites and transcript boundaries that are supported by RNA-Seq evidence from candidates that are more likely to reflect transcriptional noise, incomplete RNA processing, alignment artifacts, or weak background transcription. Figure 1 illustrates the central challenge: RNA-Seq coverage contains changes in coverage that correspond to transcript starts, transcript ends, and splice sites, but not every observed junction or coverage transition reflects a reliable transcript feature. Some spliced reads produce little or no corresponding coverage changepoint and may represent transcriptional or alignment noise.

SpliceCOV is designed to filter these noisy RNA-Seq-derived candidate features. It uses LightGBM classifiers trained on coverage- and junction-derived features to assign confidence scores to candidate transcriptional features. LightGBM was selected because it is well-suited to classification tasks involving the large, structured datasets typical of genome-wide RNA-Seq. Gradient-boosted decision trees (GBDTs) [25], of which LightGBM is a scalable implementation, are particularly effective for tabular data and can capture nonlinear relationships among coverage- and junction-derived features while remaining computationally efficient. We chose this approach as a strong and practical classifier for evaluating the proposed feature set, rather than using an automated model-selection framework to search across many model classes. Conventional GBDT implementations require scanning all instances across all features, making them computationally burdensome at nucleotide resolution. LightGBM uses histogram-based split finding and includes optimizations such as Gradient-based One-Side Sampling (GOSS) and Exclusive Feature Bundling (EFB), which provide substantial speedups and memory savings with minimal loss in accuracy.

The SpliceCOV workflow differs between splice-site and TSS/TES candidate filtering because the available candidate evidence differs between these tasks. For splice-site filtering, candidate sites are obtained directly from the splice-junction input file. Each observed junction contributes two candidate splice sites, corresponding to its donor and acceptor coordinates. These candidate sites are passed directly to a trained LightGBM classifier after feature extraction. The observed junction set provides a sensitive baseline candidate pool, and the main task is to classify each candidate as likely real or likely noisy.

For TSS/TES identification, RNA-Seq alignments do not yield a candidate set analogous to the one obtained from splice junctions. SpliceCOV therefore uses a coverage-based heuristic to propose candidate TSS/TES positions from directional changes in RNA-Seq coverage. These candidate boundaries are then filtered using a separate LightGBM classifier trained on local coverage- and junction-derived features. In this part of the workflow, coverage transitions are used only to generate candidate transcript boundaries; the final prediction step remains supervised filtering.

**Figure 2:**
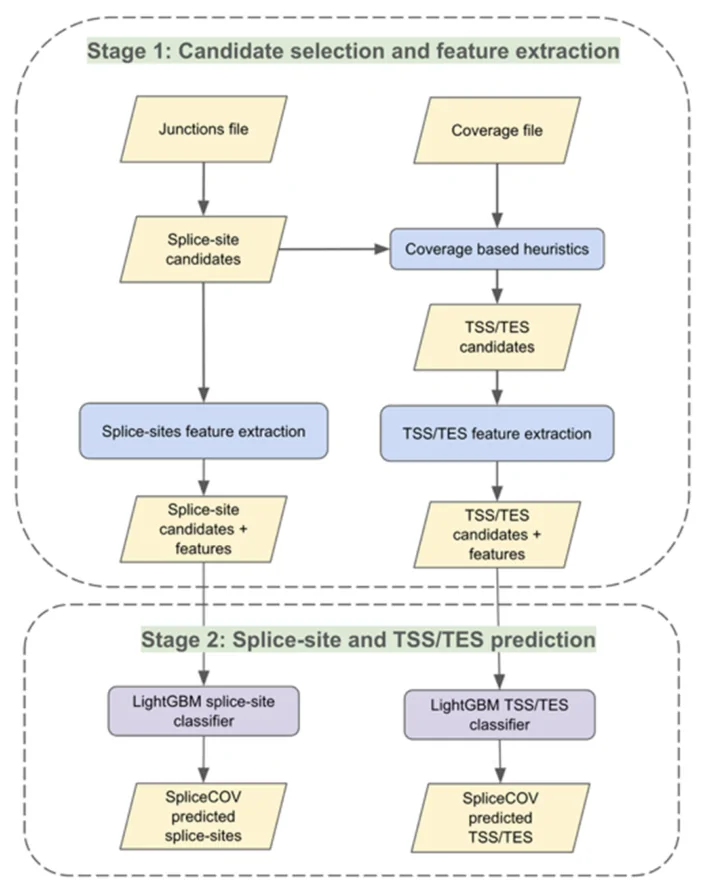
Workflow of the SpliceCOV pipeline for filtering splice-sites and TSS/TES candidates. The workflow diagram illustrates the steps of the SpliceCOV pipeline. Either single-sample or multi-sample coverage and junction files can be used as input. When multiple single-sample files are available, we recommend first preprocessing them with TieBrush and TieCov. The resulting coverage and junction data are then preprocessed and used for downstream feature extraction. Feature extraction is then performed on the candidate TSS/TES and splice sites. These features are fed into two SpliceCOV classifiers: one for TSS/TES prediction and another for splice site prediction, resulting in the final predicted sites.

A general overview of the SpliceCOV workflow is shown in Figure 2. Either single-sample or multi-sample coverage and junction files can be used as input. When multiple RNA-Seq samples are available from the same tissue or condition, we first aggregate them with TieBrush and TieCov to obtain a non-redundant junction file and a genome-wide coverage track. Splice-site candidates are extracted directly from the junction file, while TSS/TES candidates are generated from the coverage track. Candidate features are then scored by task-specific LightGBM classifiers, resulting in filtered splice-site and transcript-boundary predictions.

### 2.2. Input Requirements and Preprocessing

SpliceCOV requires two primary inputs: (i) a splice-junction file (in BED format) listing candidate donor–acceptor pairs with supporting read counts, and (ii) a genome-wide coverage track (in bigWig format) reporting per-base RNA-Seq read depth. Each input can be derived from a single RNA-Seq sample, or optionally, aggregated across multiple biologically or technically related samples, such as replicates from the same tissue, so that coverage patterns remain consistent.

To generate these inputs, we used TieBrush and TieCov [26]. TieBrush produces a splice-junction file by consolidating splice sites observed across one or more RNA-Seq samples, resulting in a non-redundant set of candidate donor–acceptor pairs with supporting read counts. TieCov generates a genome-wide coverage track by merging alignments from one or more samples to compute per-base read depth, producing an aggregated coverage profile across the input samples.

### 2.3. Splice-Site Candidate Definition and Feature Extraction

To classify candidate splice sites, SpliceCOV extracts local coverage features around each splice site coordinate derived from aggregated TieBrush junctions. Each junction contributes two candidate splice sites, corresponding to the donor and acceptor coordinates, which are treated independently. For each splice site, we extract a ±5 nt window centered on the splice site and compute six coverage- and junction-derived features:

(i) number of junction reads supporting the splice site;
(ii) absolute coverage difference between one base upstream and downstream of the splice site;
(iii) percentage coverage difference relative to the local background, defined as the absolute coverage difference divided by the mean coverage in the window;
(iv) junction length, defined as the genomic distance between donor and acceptor sites;
(v) a smoothness metric capturing the sharpness of the coverage transition, computed as the ratio between the coverage change at the splice site and the second-largest absolute change within the window; and
(vi) a binary strand-consistency flag indicating whether the direction of the coverage change is consistent with the inferred transcriptional strand from junction orientation.

These features are designed to capture RNA-Seq-specific evidence that distinguishes supported splice sites from likely noisy junctions. To evaluate whether the features separated annotated from non-annotated candidates, we performed kernel density estimation separately for candidate splice sites that matched CHESS v.3.1.3 annotations [27] and candidate splice sites that did not match CHESS annotations across three representative GTEx tissues (bladder, small intestine, thyroid; Supplementary Figure S1). We note that non-annotated candidates may include genuine splice sites absent from the reference annotation. Annotated versus non-annotated status was therefore used as a practical proxy for splice-site support rather than as definitive biological truth. This approximation is useful in human RNA-Seq because reference annotations capture a large fraction of well-supported splice sites, although some non-annotated candidates may still represent genuine novel events [28,29]. Continuous features were quantile-transformed for visualization because the raw features span different numerical ranges. These KDE analyses were used for feature assessment and visualization, not for model fitting. Across tissues, annotated splice sites showed stronger coverage contrast, sharper local transitions, and characteristic junction-length distributions compared with non-annotated candidates, supporting the use of these features as locus-agnostic predictors. To assess the contribution of individual classifier features, we calculated LightGBM gain-based feature importance, defined as the total reduction in the training objective contributed by splits using each feature. Feature importance values were normalized to sum to 100%.

### 2.4. TSS/TES Candidate Generation and Feature Extraction

Unlike splice sites, TSS/TES are not directly provided by the splice-junction input file. SpliceCOV therefore first generates candidate TSS/TES positions from the RNA-Seq coverage profile. Candidate boundary detection is performed within expressed contiguous genomic regions, defined as coverage-supported regions at least 150 bp in length and containing a contiguous segment with an average coverage greater than 10 reads. Within these expressed regions, coverage is evaluated using 150 bp windows. Positions showing a strong directional change in coverage are retained as candidate transcript boundaries, with a strong local change defined as a coverage ratio below 0.2 across the window. To reduce redundant calls, only the strongest change within each 150 bp neighborhood is retained. The numerical parameters used during TSS/TES candidate generation were selected empirically during method development based on an inspection of RNA-Seq coverage profiles. Specifically, the 150 bp region and neighbourhood sizes were chosen to capture local transcript-boundary changes; the mean-coverage threshold of 10 was used to exclude very low-coverage regions; the coverage-ratio threshold of 0.2 was used to identify pronounced local coverage changes; the 25 bp splice-site exclusion was used to reduce confusion between transcript boundaries and nearby splice-associated coverage changes; and the ±50 bp feature window was used to summarize local coverage behavior around each candidate site. These values were applied consistently across all tissues and experiments.

Candidate transcript boundaries located within 25 bp of candidate splice sites are removed before classification. This step reduces misclassification of splice-associated coverage transitions as TSS/TES. The remaining candidate sites are then treated as putative TSS/TES positions and passed to the TSS/TES LightGBM classifier.

For each candidate site, we compute an initial set of seven coverage-derived features used as input to the classifier:

(i) raw coverage at the TSS/TES site;
(ii) local mean coverage, defined as the average coverage in the ±50 bp window centered on the candidate site, normalized by the maximum local mean coverage observed in the training set;
(iii) absolute coverage difference across the boundary, measuring the magnitude of the coverage change between the upstream and downstream sides of the candidate site;
(iv) slope angle, defined as the angle (in degrees) of a linear regression fit to the coverage values within a ±50 bp window centered on the candidate site;
(v) coverage slope, given by the regression coefficient of the same linear fit, capturing the direction and steepness of the coverage transition;
(vi) a smoothness metric capturing the sharpness of the coverage transition, computed from normalized first differences of coverage within the window, with lower variability indicating cleaner, less noisy transitions; and
(vii) a binary direction-consistency flag indicating whether the direction of the coverage change matches biological expectations (increasing coverage for TSS and decreasing coverage for TES), encoded as a categorical feature prior to model training.

All numeric features are provided directly to LightGBM, while the direction-consistency indicator is encoded as an integer-valued categorical feature.

### 2.5. Model Training

SpliceCOV trains separate LightGBM binary classifiers for splice-site filtering and TSS/TES filtering. In SpliceCOV, LightGBM functions strictly as a supervised classifier. It does not perform segmentation and does not define candidate splice sites. For splice sites, candidates are obtained directly from observed junctions; for TSS/TES, candidates are generated by the coverage-based heuristic described above.

All models were trained on features derived from aggregated GTEx spleen RNA-Seq (*n* = 118). Spleen was selected as the training tissue because it provided substantial sample depth while allowing model generalization. Candidate sites overlapping CHESS annotations were labeled as positives, while candidates not overlapping CHESS annotations were labeled as negatives. For splice-site classification, positives were defined as candidate donor or acceptor sites matching CHESS-annotated splice sites, and negatives were defined as observed junction-derived candidate sites not matching CHESS. We recognize that this negative class may include genuine but currently unannotated splice sites or transcript boundaries. Therefore, these labels should be interpreted as proxies for annotation support rather than definitive biological truth. For TSS/TES classification, positives were candidate boundaries located within the specified window around annotated CHESS transcript start or end sites, and negatives were candidate boundaries outside the annotation-matching window.

For each task, candidate sites were split into stratified training and validation sets using an 80/20 split. Models were optimized using binary log-loss, with early stopping after 50 rounds without improvement in validation loss. Because negative candidates substantially outnumber positive candidates, class imbalance was handled separately for the two tasks. For splice-site classification, all candidates were derived from observed splice junctions, including annotation-negative candidates supported by junction-spanning reads. We therefore retained the full splice-site candidate set and used class weighting to account for the imbalance. In contrast, TSS/TES candidates were generated from local coverage transitions, which produced a much larger and more redundant set of annotation-negative candidates, often consisting of multiple nearby candidates associated with the same local coverage fluctuation. To reduce this redundancy and prevent the negative class from dominating training, TSS/TES negatives were randomly subsampled to match the number of positives, using random seed 42. Also, the candidate-level 80/20 split was used only for model development, including validation-loss monitoring, model selection, and early stopping. Because nearby candidate sites, including donor and acceptor sites from the same junction, can occur in different partitions, this split was not treated as an independent test of genomic generalization. Genomic generalization was instead assessed using the chromosome-holdout experiment described in Section 2.10, which supersedes the candidate-level validation split for this purpose.

All classifiers were trained with a learning rate of 0.05, 31 leaves per tree, and up to 1000 boosting rounds. Model selection was determined by validation performance, and inference was performed using the best iteration selected by early stopping. The trained models output a probability score between 0 and 1 for each candidate site. Unless otherwise specified, candidates were called positive using a fixed probability threshold of 0.4 for both splice sites and TSS/TES. This threshold is adjustable through command-line arguments.

### 2.6. Evaluation on GTEx RNA-Seq Data

To evaluate SpliceCOV, we applied TieBrush and TieCov to GTEx v8 RNA-Seq data [23] to generate per-tissue aggregated junction files and coverage tracks. These aggregated files were provided to SpliceCOV for splice-site filtering and TSS/TES prediction. Splice-site performance was evaluated by comparing tissue-aggregated TieBrush junction-derived candidate sites with CHESS v3.1.3 annotations [27]. CHESS is a filtered reference annotation and does not retain every splice site or transcript boundary present in GTEx RNA-Seq data. Genuine tissue-specific or low-abundance sites may therefore be absent from CHESS, while CHESS may contain sites supported by other tissues or external reference annotations that are not observed in a particular GTEx tissue. Candidate splice sites overlapping CHESS-annotated splice sites were counted as true positives, while candidate splice sites not overlapping CHESS annotations were counted as false positives. Annotated CHESS splice sites present in the baseline junction-derived candidate set but not retained by the method were counted as false negatives, and true negatives were baseline candidates that did not overlap CHESS annotations and were filtered out by the method. Because some non-annotated junctions may represent genuine unannotated transcriptional events, these labels should be interpreted as annotation-based proxies rather than absolute truth. Accuracy was computed only within the baseline junction-derived candidate set and should therefore be interpreted as an annotation-based candidate-set metric; sensitivity, precision, and F1 score were used as the primary comparison metrics.

To assess whether splice-site evaluation depended on the use of CHESS as the reference annotation, we repeated the bladder and small-intestine analyses using three independent human transcript annotations: MANE Select v1.5, the curated RefSeq NM/NR transcript set from RefSeq release RS_2025_08, and the complete RefSeq annotation from the same release. Only the reference annotation used to define annotation-supported and non-supported splice sites was changed. The SpliceCOV model was not retrained for these analyses.

For TSS/TES evaluation, there is no direct equivalent to the splice-junction-derived baseline used for splice sites. Predicted transcript boundaries were therefore compared directly with CHESS transcript annotations. Detecting TSS/TES is inherently more challenging than splice-site detection, because RNA-Seq coverage near transcript termini is often affected by read-through transcription, incomplete RNA degradation, and library-specific biases [30,31]. When evaluating TSS/TES filtering accuracy, because RNA-Seq coverage alone does not permit precise single-nucleotide localization of TSS/TESs, a predicted changepoint was considered overlapping if it occurred within 50 bp of a CHESS-annotated TSS/TES. Because a tissue-specific recall denominator is not straightforward to define for TSS/TES detection in RNA-Seq data, we additionally report a conservative denominator-based sensitivity estimate using the full set of 131,927 CHESS-annotated transcript start and end positions genome-wide. Sensitivity was calculated as the number of CHESS-annotated TSS/TES positions recovered by each method divided by 131,927, while precision was calculated as the fraction of predicted TSS/TES positions overlapping CHESS annotations within the same 50 bp window. Because this denominator includes transcript boundaries from genes that may not be expressed in the evaluated tissue, these sensitivity values should be interpreted as lower-bound genome-wide estimates rather than tissue-specific recall.

To evaluate the effect of sequencing depth on SpliceCOV performance, we performed a controlled downsampling analysis using the aligned RNA-Seq data from bladder and small intestine. Reads were randomly subsampled to retain 10%, 25%, 50%, 75%, or 100% of the original aligned reads. Sampling was performed by read name so that paired-end mates and alternative alignments from the same read were retained together. For each downsampling level, junction and coverage inputs were regenerated and analyzed using the same trained SpliceCOV model and decision threshold. Splice-site predictions were evaluated against the same reference annotation and using the same sensitivity, precision, and F1-score definitions as in the main analysis.

### 2.7. Comparison with Changepoint and Segmentation Methods

We evaluated established changepoint and segmentation methods as comparison baselines for RNA-Seq transcriptional feature detection. These comparisons were designed to test how well changepoints produced by general segmentation methods correspond to RNA-Seq-derived transcriptional features under the same annotation-based evaluation framework. They were not intended to evaluate the general usefulness of segmentation methods outside this specific RNA-Seq filtering task.

For splice-site analyses, we compared SpliceCOV with PELT as implemented in the Python package ruptures [32], Segmentor3IsBack, and GFPOP. PELT uses dynamic programming with pruning to optimize a penalized changepoint objective. GFPOP uses functional pruning to improve the efficiency of exact penalized segmentation with graph-constrained changepoint models. For TSS/TES analyses, we compared SpliceCOV with PELT, Segmentor3IsBack, and DeCAFS, which is designed to detect changes in signals with local fluctuations and autocorrelated noise. As an additional simple baseline for splice-site filtering, we filtered the TieBrush junction set by read support. Junctions supported by more than five reads were retained, and their corresponding donor and acceptor sites were evaluated using the same annotation-based framework applied to SpliceCOV and the segmentation methods. This analysis tests whether a simple junction-support cutoff can reproduce the improvements obtained with SpliceCOV without using local coverage-derived features.

Parameters for comparison methods were selected based on preliminary grid searches to produce biologically plausible changepoint densities and to avoid extreme under- or over-segmentation. The same fixed parameter settings were then applied across tissues for each method. PELT was run on tissue-level bedGraph coverage using an L2 cost function, a penalty of 20,000, minimum segment size of 100 bp, jump size of 5 bp, and chunk size of 2000 bp. Segmentor3IsBack was run on 1 Mb chunks using 20 bp bins, model 2, four labels, and a target density of 374 changepoints per Mb. GFPOP was run using 5 bp binned coverage on 100 kb chunks with beta = 1, gap = 0.25, and K = 2 sigma. DeCAFS was run using 50 bp bins and 1 Mb chunks, beta multipliers of 1, a minimum absolute delta of 0.3, and unstranded mode.

To assess sensitivity to the selected operating points, we additionally varied the principal stringency parameter of each segmentation method while holding the remaining settings fixed. We swept the PELT penalty, GFPOP beta, Segmentor3IsBack target changepoint density, and DeCAFS beta across broad ranges and evaluated the resulting sensitivity–precision trade-offs on small-intestine chromosome 11 (Supplementary Figure S2).

Because general segmentation methods do not label changepoints by feature type, we used CHESS annotations only for evaluation-time assignment of changepoints to splice-site or transcript-boundary categories. Specifically, changepoints within 100 bp of an annotated splice junction were evaluated in the splice-site category, whereas remaining changepoints were evaluated in the TSS/TES category. This annotation-informed assignment is not intended as a deployable classification rule and was used only to enable comparison under a common evaluation framework.

### 2.8. Integration of SpliceCOV Predictions into Transcriptome Assembly

To evaluate whether SpliceCOV-filtered features improve downstream transcriptome assembly, we incorporated predicted splice sites and transcript-boundary positions into StringTie using the StringTie point-feature input option. For each sample, StringTie v3.0.1 was run in two configurations: a baseline configuration using its default assembly procedure without external point features, and in a SpliceCOV-guided configuration using the ‘--ptf’ option with SpliceCOV-filtered splice sites and SpliceCOV-predicted TSS/TES positions as external guidance.

All StringTie assemblies were compared against reference annotations using gffcompare [33]. Transcript-level matching followed gffcompare’s standard criteria. For multi-exon transcripts, this corresponds to a complete intron-chain match to a reference transcript, with the same number of introns and identical donor and acceptor coordinates for each intron, while exact agreement of transcript start and end coordinates is not required. For single-exon transcripts, gffcompare uses overlap-based matching criteria, requiring at least 80% reciprocal overlap between the assembled transcript and the reference transcript. Transcript-level precision and the number of reference-matching transcripts reported by gffcompare were used as the primary evaluation metrics. For each tissue, the statistical significance of the change in precision between default and SpliceCOV-guided assemblies was assessed using a paired *t*-test, with assemblies paired by RNA-Seq sample. Tests were paired by RNA-Seq sample because each sample was assembled once with default StringTie and once with point-feature guidance.

### 2.9. Cross-Species Application to Mouse RNA-Seq

To assess cross-species generalization, SpliceCOV was applied to mouse RNA-Seq data from ENCODE using the model trained on human GTEx spleen data [34]. The mouse analysis used pre-aligned ENCODE BAM files from seven ENCODE datasets: ENCSR703ACF, ENCSR788PEE, ENCSR243UCH, ENCSR092BBW, ENCSR034MGF, ENCSR377UPT, and ENCSR968ZOA. Mouse splice-site calls were evaluated against the GENCODE mouse annotation for GENCODE vM10/GRCm38 using the same framework used for human tissues [35]. TieBrush junctions served as the baseline candidate set, and SpliceCOV directly classified candidate donor and acceptor sites derived from these observed junctions. Similar to the previous evaluation, candidate sites overlapping GENCODE-annotated splice sites were counted as true positives, non-overlapping candidate sites were counted as false positives, annotated splice sites present in the baseline candidate set but not retained by SpliceCOV were counted as false negatives, and non-overlapping baseline candidates filtered out by the method were counted as true negatives. Because some non-GENCODE candidate sites may represent genuine but currently unannotated splice sites, these labels should be interpreted as annotation-based proxies rather than absolute truth.

To evaluate whether cross-species filtering improved downstream assembly, StringTie was run on the mouse RNA-Seq data with and without SpliceCOV-filtered features. Assembly outputs were compared against the GENCODE mouse annotation using the same transcript-level metrics applied in the human analyses. This experiment tested whether the trained LightGBM model captured generalizable RNA-Seq evidence for splice-site support rather than human-specific genomic loci. Differences between baseline and SpliceCOV-guided mouse assemblies were assessed using paired *t*-tests, with assemblies paired by RNA-Seq sample.

For the assembly comparison, paired *t*-tests were performed across 20 matched BAM/library observations from the seven ENCODE datasets, with each BAM assembled once using default StringTie and once using SpliceCOV guidance (*n* = 20 pairs; df = 19). More information on the mouse ENCODE dataset could be found in Supplementary Figure S3.

### 2.10. Chromosome-Holdout Evaluation

To test whether SpliceCOV learned generalizable coverage-based features rather than memorizing genomic loci present in the training data, we performed a chromosome-holdout evaluation. For this experiment, SpliceCOV models were trained using candidate features from GTEx spleen data on chromosomes 2, 4, 6, 8, 10–22, X, and Y. Chromosomes 1, 3, 5, 7, and 9 were excluded from training and used only for evaluation. The same feature definitions, labeling strategy, model hyperparameters, and class-imbalance handling described above were used for the holdout-trained models.

The chromosome-holdout models were evaluated on held-out chromosomes in two representative GTEx tissues, bladder and small intestine. For splice-site filtering, candidate donor and acceptor sites were derived from the TieBrush junction files, restricted to chromosomes 1, 3, 5, 7, and 9, and evaluated against CHESS-annotated splice sites using the same annotation-based definitions of true positives, false positives, false negatives, and true negatives described above.

For TSS/TES evaluation, candidate transcript-boundary positions were generated from coverage on the held-out chromosomes and scored using the chromosome-holdout TSS/TES classifier. Predicted TSS/TES positions were compared with CHESS-annotated transcript start and end sites using the same 50 bp matching window used in the main TSS/TES evaluation. TSS and TES positions were pooled for this analysis. Performance of the chromosome-holdout model was compared with that of the model trained using all chromosomes. Segmentation-based comparison methods were evaluated on the same held-out chromosomes using the same parameter settings as in the genome-wide analyses.

### 2.11. Software Versions

The main software versions used for the analyses were TieBrush v0.0.7, TieCov v0.0.6, StringTie v3.0.1, gffcompare v0.12.6, Python v3.9.19, R v4.2.3, LightGBM v4.4.0, ruptures v1.1.10, gfpop R package v1.1.1, Segmentor3IsBack v2.0, and DeCAFS v3.3.5.

Segmentor3IsBack v2.0 is no longer available through the active CRAN repository. For the reproduced benchmarks, we obtained the archived source package Segmentor3IsBack_2.0.tar.gz from the CRAN archive and compiled and installed it using R 4.1.3 via R CMD INSTALL into a dedicated R library. All Segmentor3IsBack analyses in these reproduced benchmarks used this archived v2.0 installation.

## 3. Results

We applied SpliceCOV to GTEx v8 RNA-Seq data across 30 tissue/group aggregates using per-tissue aggregated splice-junction and coverage tracks generated by TieBrush and TieCov. Spleen was the training tissue and was therefore not treated as an independent test of cross-tissue generalization, although spleen results are shown in some tissue-level summaries for illustrative comparison. Predictions were evaluated against CHESS v3.1.3 annotations.

### 3.1. Splice-Site Filtering Accuracy in GTEx Tissues

**Figure 3:**
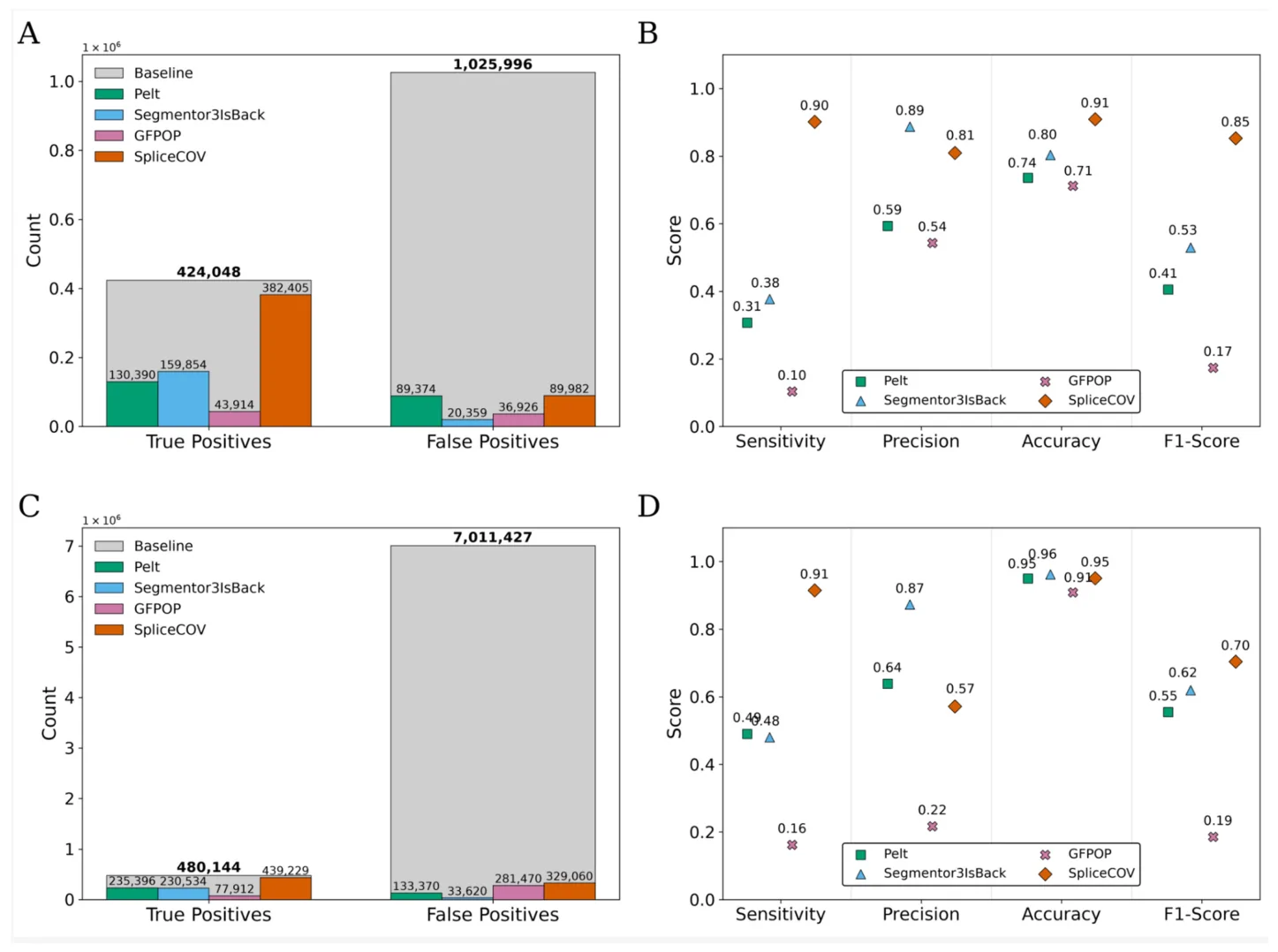
Performance of splice site identification on multi-sample aggregated RNA-Seq coverage in bladder and small intestine. Evaluation of splice site filtering using aggregated RNA-Seq coverage, comparing PELT, Segmentor3IsBack, GFPOP, and SpliceCOV. (**A**) Counts of true positives (TP) and false positives (FP) for bladder samples, relative to Baseline (the set of all splice sites inferred from RNA-Seq alignments). (**B**) Corresponding performance metrics for bladder samples, including sensitivity, precision, accuracy, and F1 score. (**C**) Counts of true positives (TP) and false positives (FP) for small intestine samples. (**D**) Corresponding performance metrics for small intestine samples, including sensitivity, precision, accuracy, and F1 score. Colors indicate methods as shown in the legend.

We first evaluated splice-site filtering in two representative tissues with different sample depths: bladder (11 samples) and small intestine (104 samples). In each tissue, the unfiltered TieBrush junction endpoints were used as the baseline candidate set. In bladder, the baseline contained 424,048 annotated splice sites and 1,025,996 non-annotated candidates. SpliceCOV retained 382,405 annotated splice sites, corresponding to a sensitivity of 0.90, while reducing putative false positives to 89,982, a 91.2% reduction relative to the baseline. This yielded a precision of 0.81 and the highest F1-score among the evaluated methods, 0.85. By comparison, PELT and Segmentor3IsBack retained only 30.7% and 37.7% of annotated splice sites, respectively. GFPOP recovered the fewest annotated splice sites (43,914) and achieved a moderate precision of 0.54. Segmentor3IsBack achieved the highest precision, 0.89, but at the cost of much lower sensitivity and an F1-score of 0.53 (Figure 3A,B). Because SpliceCOV is supervised using annotation-derived labels whereas the comparison methods are unsupervised segmentation algorithms, these results should be interpreted as task-specific behavior under the RNA-Seq filtering framework rather than as a general ranking of the methods. For the segmentation baselines, generic changepoints were assigned to splice-site or TSS/TES categories using annotation-assisted mapping so that they could be evaluated within the same feature-specific framework. SpliceCOV predictions were generated directly as splice-site or transcript-boundary scores and were therefore not subjected to this additional assignment step.

An additional distinction is that the segmentation methods return generic changepoints and do not intrinsically assign them to splice-site or transcript-boundary categories. For these methods, CHESS annotations were therefore used only at evaluation time to assign changepoints to the corresponding feature category, providing an annotation-assisted framework for comparison. SpliceCOV predictions were not computed using this assignment rule. Splice-site candidates in SpliceCOV were defined directly from observed junction donor and acceptor coordinates, whereas TSS/TES candidates were generated separately from RNA-Seq coverage before classification.

A similar pattern was observed in the small intestine. The baseline contained 480,144 annotated splice sites and 7,011,427 non-annotated candidates. SpliceCOV retained 439,229 annotated splice sites, corresponding to a sensitivity of 0.91, while reducing putative false positives to 329,060, a 95.3% reduction relative to the baseline. This yielded a precision of 0.57 and an F1 score of 0.70. Although Segmentor3IsBack and PELT achieved higher precision, 0.87 and 0.64, respectively, they retained less than half of the annotated splice sites. GFPOP was the most conservative in true-site recovery, with a sensitivity of 0.16 (Figure 3C,D).

To determine whether these results depended on CHESS as the evaluation reference, we repeated the splice-site analysis using MANE Select v1.5, RefSeq NM/NR, and the complete RefSeq annotation (Supplementary Figure S4). The overall results were consistent across reference annotations. Using RefSeq NM/NR, SpliceCOV achieved a sensitivity, precision, and F1 score of 0.92, 0.77, and 0.84 in bladder and 0.94, 0.53, and 0.68 in small intestine, compared with 0.90, 0.81, and 0.85 and 0.91, 0.57, and 0.70, respectively, using CHESS. Using the complete RefSeq annotation, SpliceCOV achieved sensitivity, precision, and F1 scores of 0.88, 0.82, and 0.85 in bladder and 0.90, 0.59, and 0.71 in small intestine. These analyses indicate that the main splice-site filtering results were not specific to CHESS as the reference annotation.

**Figure 4:**
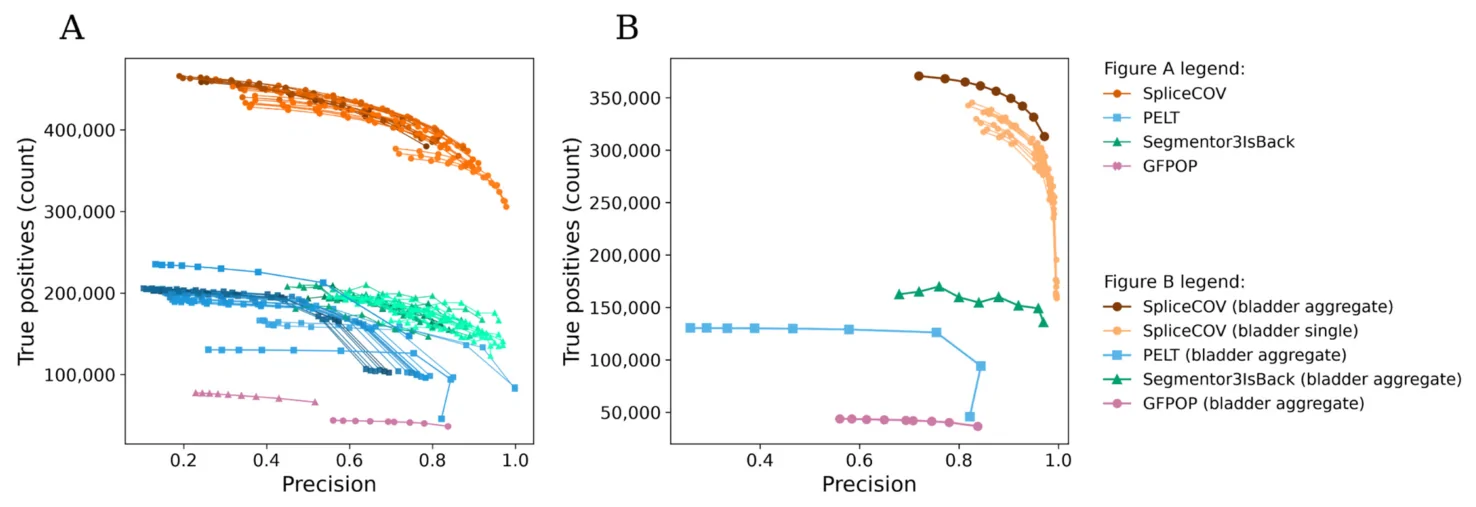
Precision-true positive trade-off for splice site detection. (**A**) Performance across all 30 GTEx tissues when RNA-Seq coverage is aggregated from all samples in a tissue. Each curve corresponds to a GTEx tissue and depicts the number of true positives (*y*-axis) versus precision (*x*-axis) as the confidence-score threshold varies. Lighter shades represent tissues with fewer samples. GFPOP results are shown only for bladder and small intestine because whole-genome GFPOP analyses are computationally intensive and were therefore completed only for these two representative tissues. (**B**) SpliceCOV performance (in orange) on each of the 11 individual bladder samples. For comparison, results on the aggregated bladder dataset for SpliceCOV (brown), PELT (blue), Segmentor3IsBack (green), and GFPOP (pink) are also shown.

To evaluate how SpliceCOV generalizes beyond the two tissues examined above, we applied the spleen-trained model to all 30 GTEx tissues, which include bladder and small intestine and span a wide range of samples, ranging from fewer than a dozen to over one thousand per tissue. For each tissue, we plotted true-positive counts against precision at varying confidence thresholds (Figure 4A). Across all tissues, SpliceCOV consistently recovered more true positives at equivalent precision levels compared to PELT and Segmentor3IsBack. The three lighter curves in the figure correspond to tissues with fewer than 12 samples. In these small-sample tissues, SpliceCOV achieved higher precision across all thresholds, but recovered fewer true positives compared to tissues with a higher number of samples, reflecting their smaller baseline junction sets. Accordingly, for tissues with a limited number of samples (fewer than 20), we recommend applying a lower confidence threshold when filtering out potential noisy splice sites, in order to maximize true splice-site detection.

To assess whether the SpliceCOV model trained on aggregated spleen data also performs well on individual RNA-Seq samples, we evaluated its performance on single-sample coverage profiles. Figure 4B shows that SpliceCOV maintains consistent behavior across individual bladder samples. When applied to single samples, the model still recovered more true splice sites at comparable precision levels than PELT or Segmentor3IsBack, although the overall signal was weaker than in aggregated datasets. We therefore recommend a threshold of 0.3 for datasets containing fewer than 10 aggregated RNA-Seq samples and the default threshold of 0.4 for datasets containing 10 or more samples. In the low-sample datasets, lowering the threshold from 0.4 to 0.3 increased sensitivity from approximately 0.70 to 0.75 while decreasing precision from approximately 0.95 to 0.90, illustrating the expected sensitivity–precision trade-off.

We next evaluated the effect of sequencing depth by randomly downsampling the aligned RNA-Seq reads from bladder and small intestine to 10%, 25%, 50%, and 75% of the original depth (Supplementary Figure S5). SpliceCOV remained highly precise across all downsampling levels, but sensitivity decreased as sequencing depth was reduced. In bladder, sensitivity decreased from 0.91 at full depth to 0.62 at 10% depth, while precision remained high, increasing from 0.94 to 0.98. A similar pattern was observed in small intestine, where sensitivity decreased from 0.89 to 0.57 while precision remained between 0.94 and 0.97. These results indicate that reduced read depth primarily limits the recovery of annotation-supported splice sites rather than substantially increasing false-positive predictions.

We also compared SpliceCOV with a simple junction-support filter (Supplementary Figure S6). In bladder, retaining junctions supported by more than five reads achieved the same precision as SpliceCOV (0.81) but reduced sensitivity from 0.90 to 0.85, resulting in an F1 score of 0.83 compared with 0.85 for SpliceCOV. In small intestine, the same junction-support threshold retained similar sensitivity (0.91) but achieved substantially lower precision (0.43 versus 0.57), resulting in an F1 score of 0.59 compared with 0.70 for SpliceCOV. These results indicate that junction support alone can remove many weakly supported candidates, but a single fixed read-count threshold does not reproduce the same sensitivity–precision trade-off across datasets.

LightGBM gain-based feature importance further showed that the local smoothness metric accounted for 85.16% of the total gain (Supplementary Figure S7). The remaining gain was distributed across junction read support, absolute coverage difference, percentage coverage difference, junction length, and the binary strand-consistency flag. These results indicate that the classifier relies primarily on the local structure of the RNA-Seq coverage signal rather than simply reproducing a junction read-count threshold.

### 3.2. Annotation Overlap of Predicted TSS/TES in GTEx Tissues

**Figure 5:**
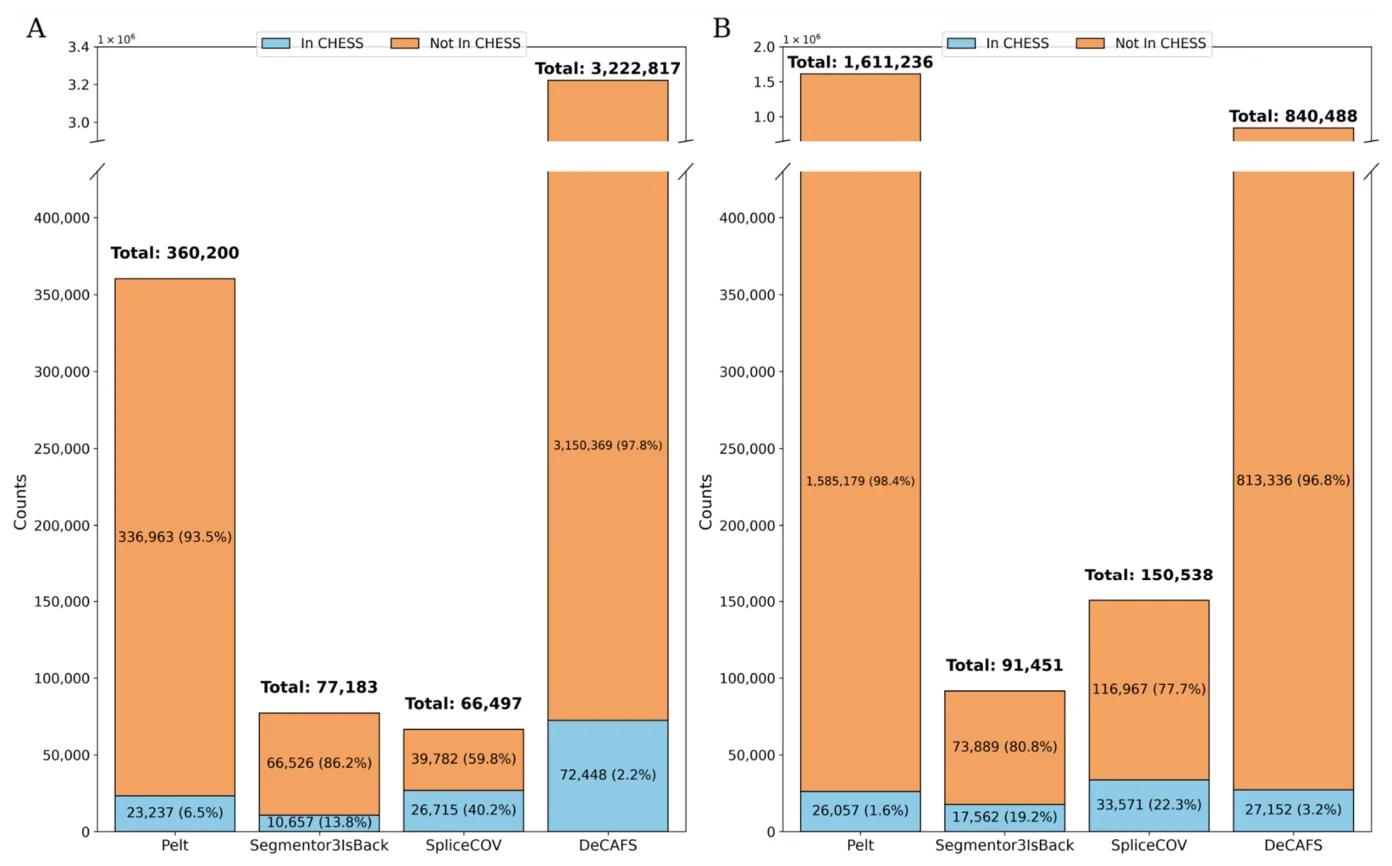
Evaluation of TSS/TES detection by PELT, Segmentor3IsBack, DeCAFS, and SpliceCOV in GTEx bladder and small intestine tissues. The stacked bars show the total number of predicted TSS/TES calls for each method (labels above each bar). Overlaps with CHESS-annotated TSS/TES are shown in blue, and not in CHESS predictions in orange. For each method, the total number of predictions is Ntotal = Nin CHESS + Nnot in CHESS. The percentages shown inside each colored segment are computed relative to that method’s total: % in CHESS = 100× (Nin CHESS/Ntotal). (**A**) Bladder dataset. (**B**) Small intestine dataset.

Unlike splice sites, which are directly supported by spliced reads, there is no obvious unfiltered baseline candidate set for TSS/TES. We therefore evaluated each method by considering both the total number of non-junction changepoints produced and the fraction and number that overlapped CHESS-annotated transcript termini. In bladder, SpliceCOV produced the fewest candidates among the evaluated methods, with 66,497 predicted TSS/TES positions, of which 26,715 overlapped CHESS annotations, corresponding to an overlap rate of 40.2%. Segmentor3IsBack produced a similar but slightly larger number of candidates, 77,183, but only 10,657 overlapped CHESS annotations, corresponding to an overlap rate of 13.8%. PELT generated 360,200 candidates, of which 23,237 overlapped CHESS annotations, for an overlap rate of 6.5%. DeCAFS produced by far the largest number of candidates, 3,222,817, including 72,448 CHESS-overlapping positions; however, 3,150,369 predictions, or 97.8%, did not overlap CHESS annotations (Figure 5A).

In small intestine, SpliceCOV again provided the strongest balance between candidate reduction and annotation overlap. It produced 150,538 candidates, of which 33,571 overlapped CHESS annotations, giving an overlap rate of 22.3%. Segmentor3IsBack produced fewer total candidates, 91,451, but had fewer CHESS-overlapping positions, 17,562, corresponding to an overlap rate of 19.2%. PELT generated 1,611,236 candidates, but only 26,057, or 1.6%, overlapped CHESS annotations. DeCAFS produced 840,488 candidates, of which 27,152, or 3.2%, overlapped CHESS annotations (Figure 5B). Overall, SpliceCOV achieved the highest CHESS-overlap rate in bladder and the largest number of CHESS-overlapping TSS/TES candidates in small intestine, while avoiding the large excess of CHESS-non-overlapping changepoints produced by PELT and DeCAFS.

To complement the annotation-overlap analysis, we also report a conservative denominator-based sensitivity estimate using the full genome-wide set of 131,927 CHESS-annotated TSS/TES positions (Supplementary Figure S8). Under this definition, SpliceCOV achieved a sensitivity, precision, and F1 score of 0.24, 0.40, and 0.30 in bladder and 0.31, 0.22, and 0.26 in small intestine. Across both tissues, SpliceCOV achieved the highest F1 score among the evaluated methods. Because the denominator includes annotated transcript boundaries from genes that are not expressed in the evaluated tissue, sensitivity values should be interpreted as lower bounds rather than estimates of true tissue-specific recall.

### 3.3. Improvement of Transcriptome Assembly Using SpliceCOV Guidance

We next evaluated whether SpliceCOV-filtered transcript features improve downstream transcriptome assembly. StringTie v3.0.1 was run on individual GTEx RNA-Seq samples either in its default configuration without point-feature guidance or with external point features supplied through the --ptf option. The SpliceCOV-guided point-feature files included SpliceCOV-filtered splice sites and SpliceCOV-predicted TSS/TES positions.

**Figure 6:**
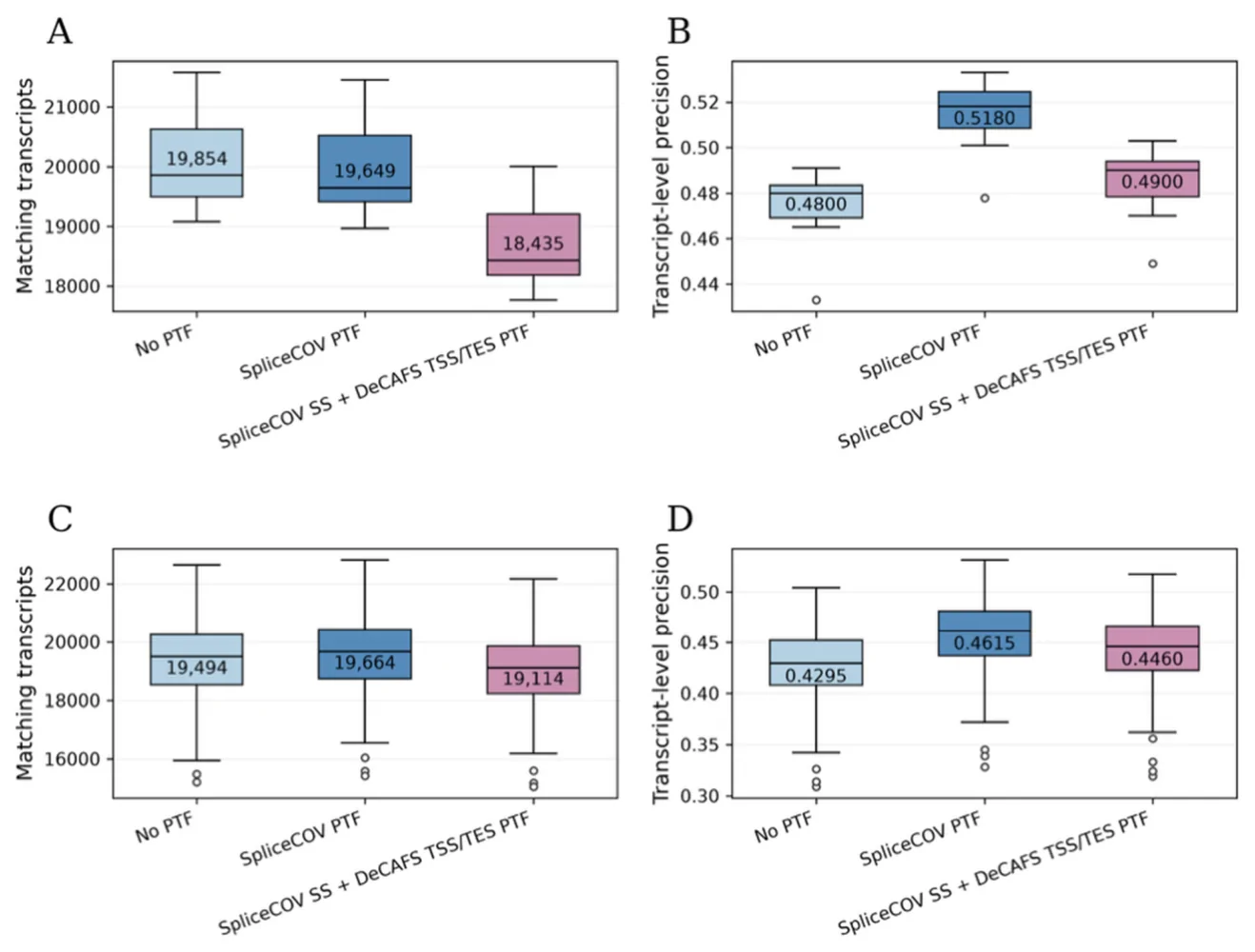

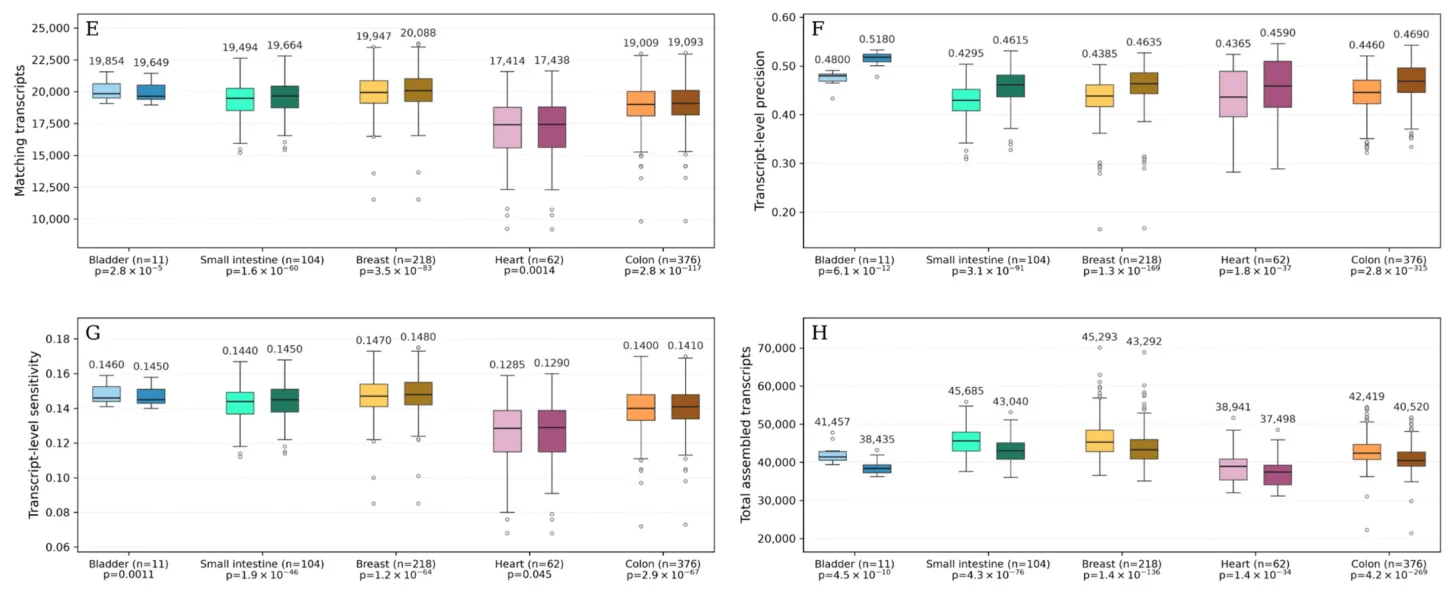
Impact of SpliceCOV-guided StringTie assembly on transcript recovery and transcript-level precision. (**A**–**D**) Effect of TSS/TES source on StringTie assembly in bladder and small intestine. StringTie was run in three configurations: default assembly without point features (“No PTF”), guidance using SpliceCOV-filtered splice sites together with SpliceCOV TSS/TES predictions (“SpliceCOV PTF”), and guidance using the same SpliceCOV-filtered splice sites together with DeCAFS-derived TSS/TES predictions (“SpliceCOV SS + DeCAFS TSS/TES PTF”). Panels show the number of reference-matching transcripts and transcript-level precision in bladder (**A**,**B**) and small intestine (**C**,**D**). (**E**–**H**) Boxplots summarize per-sample StringTie assembly results across five GTEx tissues: bladder, small intestine, breast, heart, and colon. For each tissue, default StringTie assemblies (lighter colors) are compared with assemblies guided by SpliceCOV-predicted transcript features (darker colors). (**E**) Number of reference-matching transcripts per sample. (**F**) Transcript-level precision per sample. (**G**) Transcript-level sensitivity per sample. (**H**) Total number of assembled transcripts per sample. Values shown above the boxplots indicate the median, and *p*-values below each tissue indicate the significance of paired comparisons between default and SpliceCOV-guided assemblies.

Because DeCAFS produced a larger number of CHESS-overlapping TSS/TES candidates in bladder, we first tested whether replacing SpliceCOV transcript-boundary predictions with DeCAFS-derived TSS/TES calls would further improve assembly. To isolate the effect of the TSS/TES source, we held splice-site evidence constant and compared three StringTie configurations in bladder and small intestine: (1) no point-feature guidance, (2) guidance with SpliceCOV splice sites plus SpliceCOV TSS/TES, and (3) guidance with the same SpliceCOV splice sites plus DeCAFS TSS/TES. In bladder, SpliceCOV point-feature guidance increased the median transcript-level precision from 0.48 to 0.518 while maintaining a similar number of reference-matching transcripts. In contrast, replacing SpliceCOV TSS/TES with DeCAFS TSS/TES reduced the median number of matching transcripts and achieved a lower precision than the full SpliceCOV point-feature set (Figure 6A,B). A similar pattern was observed in small intestine, where SpliceCOV guidance produced the highest median precision and slightly increased the median number of reference-matching transcripts, whereas the SpliceCOV splice-site plus DeCAFS TSS/TES configuration did not improve assembly to the same extent (Figure 6C,D). These results indicate that simply producing more annotation-overlapping TSS/TES calls does not necessarily improve transcriptome assembly, likely because excessive transcript-boundary candidates can introduce noisy evidence.

We then evaluated SpliceCOV-guided assembly across five GTEx tissues spanning a broad range of sample sizes: bladder, with 11 samples; small intestine, with 104; breast, with 218; heart, with 62; and colon, with 376. Across all five tissues, SpliceCOV-guided assemblies showed significantly higher transcript-level precision than default StringTie assemblies (Figure 6F). The number of reference-matching transcripts remained broadly similar between the default and SpliceCOV-guided assemblies, indicating that the precision gains did not come at the cost of reduced transcript recovery (Figure 6E). Together, these results show that SpliceCOV-filtered transcript features can improve StringTie assembly by reducing noisy feature evidence while preserving annotation-supported transcript structure.

### 3.4. SpliceCOV Application to Mouse RNA-Seq Data

Although SpliceCOV was trained exclusively on human spleen data, we asked whether the model would generalize to RNA-Seq data from other species. We evaluated splice site filtering on a mouse RNA-Seq dataset from the ENCODE Project, using the mouse GENCODE annotation as the reference.

**Figure 7:**
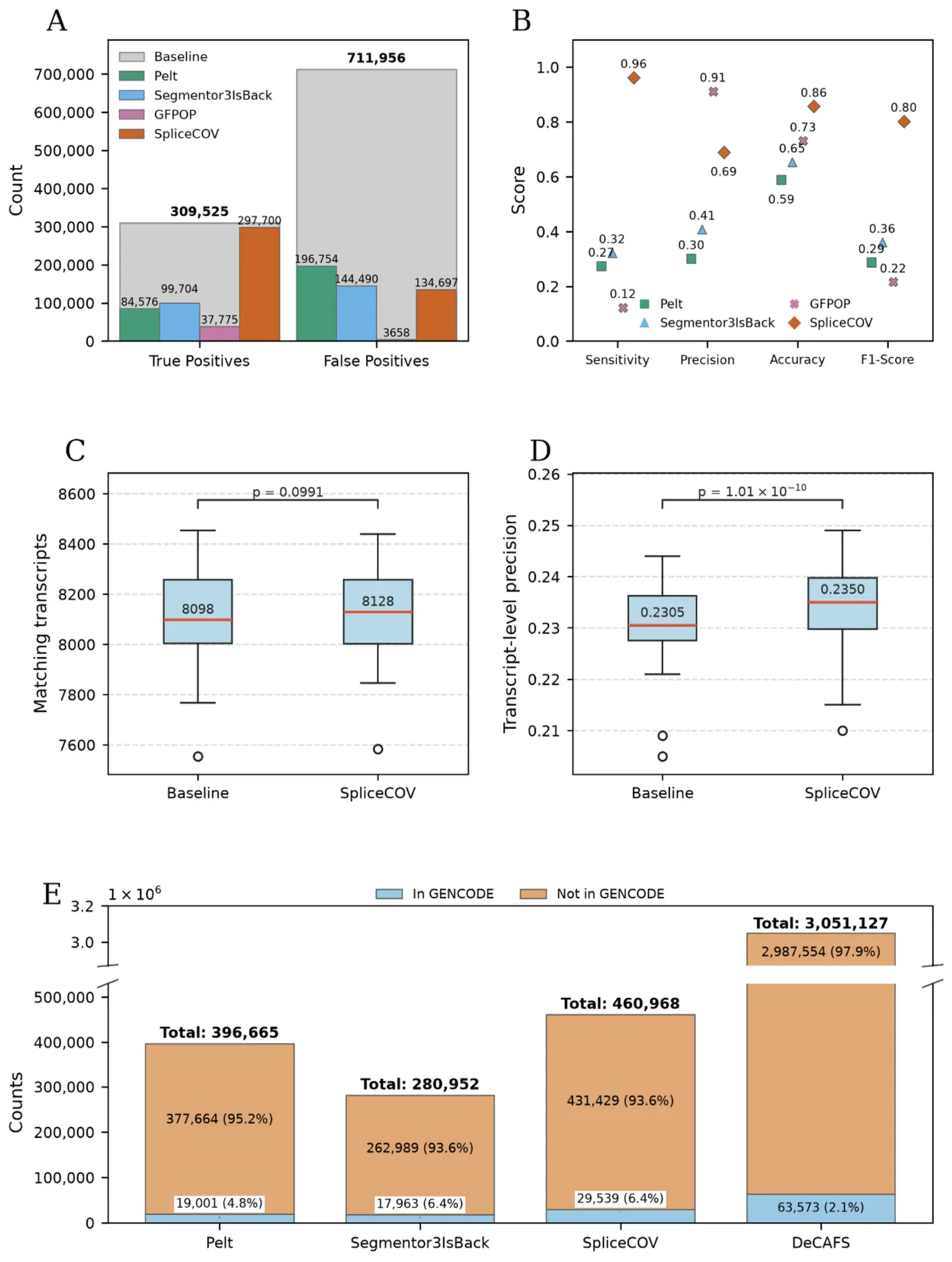
Evaluation metrics of SpliceCOV on mouse RNA-Seq. (**A**) True positives and false positives inferred from aggregated mouse RNA-Seq coverage, comparing PELT, Segmentor3IsBack, GFPOP, and SpliceCOV, relative to Baseline (the set of all splice sites inferred from the RNA-Seq alignments). (**B**) Splice site detection performance metrics, including sensitivity, precision, accuracy, and F1 score. (**C**,**D**) Impact of SpliceCOV guidance on mouse StringTie assemblies. Boxplots compare baseline versus SpliceCOV-guided runs for (**C**) number of reference-matching transcripts and (**D**) transcript-level precision. Red lines denote medians. Statistical significance was assessed using paired t-tests, with the *p*-values indicated above the boxplots. (**E**) Overlap of predicted TSS/TES with the GENCODE mouse annotation. Stacked bars show counts of predictions that fall within GENCODE (blue) versus outside GENCODE (orange), with totals and percentages annotated above each bar. For each method, the total number of predictions is N_total_ = N_in GENCODE_ + N_not in GENCODE_. The percentages shown inside each colored segment are computed relative to that method’s total: %_in GENCODE_ = 100 × (N_in GENCODE_/N_total_).

In this mouse dataset, the baseline contained 309,525 junction-derived splice-site positions overlapping GENCODE annotations, but also a large number of splice-site candidates that did not overlap the annotation, 711,956, which we classified as putative false positives (Figure 7A). PELT substantially reduced the number of putative false positives relative to the baseline to 196,754, but at the cost of sensitivity, recovering only 84,576 annotated splice sites corresponding to a recall of 0.27. This resulted in modest precision, 0.30, and an F1-score of 0.29. Segmentor3IsBack exhibited slightly higher sensitivity than PELT with 99,704 true positives and a recall of 0.32, but retained a comparable number of putative false positives, 144,490, yielding a precision of 0.41 and an F1-score of 0.36. GFPOP was the most stringent method, reducing putative false positives to only 3658 and achieving the highest precision, 0.91, but it recovered only 37,775 annotated splice sites, corresponding to a sensitivity of 0.12 and an F1-score of 0.22. In contrast, SpliceCOV retained nearly all annotated sites, with 297,700 true positives and a recall of 0.96, while reducing putative false positives more than fivefold to 134,697. This resulted in a precision of 0.69 and F1-score of 0.80 (Figure 7B). Thus, despite being trained on human data, SpliceCOV substantially increased precision while maintaining high sensitivity for junction-derived splice-site candidates in mouse RNA-Seq data.

We next asked whether these cross-species gains also translated into improved transcriptome assembly. Using the same mouse dataset, we ran StringTie in default mode and with SpliceCOV guidance, providing predicted splice sites and TSS/TES as input. SpliceCOV guidance increased the median number of reference-matching transcripts from 8098 to 8128 (Figure 7C) while slightly increasing median transcript-level precision from 0.2305 to 0.2350 (Figure 7D). These results indicate that SpliceCOV guidance can be applied to mouse RNA-Seq without retraining and can produce a modest improvement in transcriptome assembly. However, the lower transcript-level precision in mice compared with human tissues indicates that cross-species transferability is limited and that species-specific retraining may further improve performance.

For transcript termini in mouse (Figure 7E), all evaluated methods produced many TSS/TES candidates, and only a small fraction overlapped mouse GENCODE annotations, consistent with the difficulty of identifying transcript boundaries from RNA-Seq coverage alone. PELT predicted 396,665 TSS/TES candidates, of which 19,001 overlapped GENCODE, corresponding to an overlap rate of 4.8%; the remaining 377,664 candidates, or 95.2%, did not overlap the annotation. Segmentor3IsBack produced fewer candidates, 280,952 total, and achieved a slightly higher overlap rate of 6.4%, with 17,963 candidates overlapping GENCODE and 262,989 outside GENCODE. SpliceCOV generated 460,968 TSS/TES candidates, of which 29,539 overlapped GENCODE and 431,429 did not. This corresponds to an overlap rate of 6.4%, matching Segmentor3IsBack while recovering more annotated transcript termini than either PELT or Segmentor3IsBack. DeCAFS produced the largest number of GENCODE-overlapping termini, 63,573, but did so by producing 3,051,127 total candidates, of which 2,987,554, or 97.9%, were outside GENCODE.

### 3.5. Generalization to Unseen Chromosomes

To assess whether SpliceCOV’s performance reflects generalizable coverage-based features rather than the memorization of genomic loci seen during training, we performed a chromosome-holdout experiment on two representative GTEx tissues: bladder and small intestine. The SpliceCOV classifier was trained using spleen data from chromosomes 2, 4, 6, 8, 10–22, X, and Y, and evaluated on the disjoint chromosomes 1, 3, 5, 7, and 9, which were completely excluded from training.

**Figure 8:**
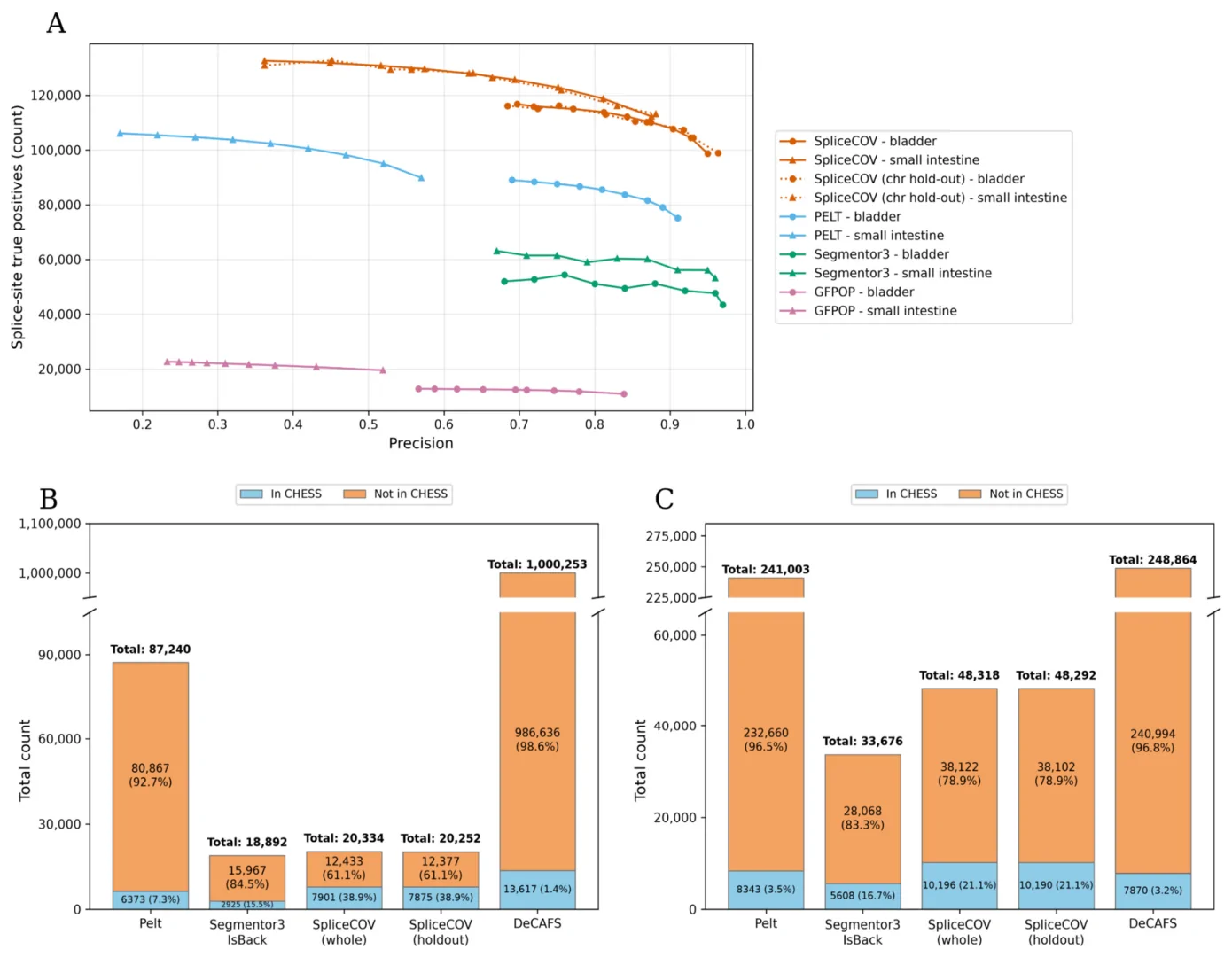
Performance comparison of splice site and TSS/TES detection in the chromosome-holdout test. All results are on chromosomes 1, 3, 5, 7, and 9. (**A**) Precision–true positive trade-off shown as the number of true positive splice sites versus precision for SpliceCOV, PELT, Segmentor3IsBack, and GFPOP in bladder (circles) and small intestine (triangles). Solid lines indicate models trained on all chromosomes, while dotted lines denote chromosome hold-out evaluation for SpliceCOV. (**B**,**C**) Overlap of predicted TSS/TES with the CHESS annotation for bladder (**B**) and small intestine (**C**). Stacked bars show counts of predictions that fall within CHESS (blue) versus outside CHESS (orange), with totals and percentages annotated above each bar. SpliceCOV (whole) refers to the model trained on transcript features on all chromosomes from the GTEx spleen samples, while SpliceCOV (holdout) refers to the model trained only on transcript features from chromosomes 2, 4, 6, 8, 10–22, X, and Y. For each method, the total number of predictions is N_total_ = N_in CHESS_ + N_not in CHESS_. The percentages shown inside each colored segment are computed relative to that method’s total: % in CHESS = 100× (N_in CHESS_/N_total_).

Figure 8A plots precision against the number of true positives obtained as the decision threshold was progressively relaxed for the held-out chromosomes compared with the genome-wide trained model. In both bladder and small intestine, the chromosome-holdout SpliceCOV curves closely matched those obtained with the genome-wide trained model, indicating minimal degradation in performance on unseen chromosomes. This similarity supports the conclusion that SpliceCOV does not rely on locus-specific memorization, but instead learns transferable local coverage signatures that generalize across genomic regions. Under the same evaluation framework, PELT, Segmentor3IsBack, and GFPOP recovered fewer annotation-supported splice sites than SpliceCOV at comparable precision levels. GFPOP showed the lowest true-positive recovery among the splice-site comparison methods, consistent with its more stringent behavior in the genome-wide splice-site evaluation.

We next evaluated whether this chromosome-holdout setup also preserved performance for TSS/TES detection. In bladder (Figure 8B), PELT produced 87,240 TSS/TES candidates, of which 6373 overlapped CHESS annotations, corresponding to an overlap rate of 7.3%. Segmentor3IsBack reduced the total number of candidates to 18,892 and achieved a higher overlap rate of 15.5%, with 2925 CHESS-overlapping sites. In contrast, SpliceCOV maintained a substantially higher CHESS-overlap rate while producing an intermediate number of calls: the genome-wide trained model produced 20,334 predictions with 7901 CHESS-overlapping sites, or 38.9%, and the chromosome-holdout model produced 20,252 predictions with 7875 CHESS-overlapping sites, also 38.9%. DeCAFS produced the largest number of candidates in bladder, 1,000,253 total, but only 13,617 overlapped CHESS, corresponding to an overlap rate of 1.4%.

A similar pattern was observed in small intestine (Figure 8C). PELT generated 241,003 candidates, of which 8343 overlapped CHESS, corresponding to an overlap rate of 3.5%. Segmentor3IsBack produced 33,676 candidates with 5608 CHESS-overlapping sites, or 16.7%. SpliceCOV again provided the strongest balance between candidate number and annotation overlap, yielding 48,318 predictions with 10,196 CHESS-overlapping sites, or 21.1%, under genome-wide training, and 48,292 predictions with 10,190 CHESS-overlapping sites, also 21.1%, under chromosome holdout. DeCAFS produced 248,864 candidates in small intestine, of which 7870 overlapped CHESS, corresponding to an overlap rate of 3.2%. Together, these results show that SpliceCOV’s splice-site and TSS/TES filtering behavior generalizes to unseen chromosomes with essentially no degradation, while maintaining a stronger balance between annotation-supported recovery and excess candidate generation than the comparison methods.

### 3.6. Speed and Memory Comparisons

We benchmarked wall-clock runtime and peak memory usage for SpliceCOV, PELT, GFPOP, and Segmentor3IsBack on GTEx tissue datasets (Supplementary Figure S9). SpliceCOV required approximately 11–173 min per tissue, whereas PELT required approximately 16–1129 min, exceeding 10 h for six tissues. Segmentor3IsBack was fastest, completing analyses in approximately 0.2–12.6 min. Because GFPOP was computationally time-consuming, we completed whole-genome runs only for bladder and small intestine, which required approximately 5.1 and 25.6 h, respectively. We expect GFPOP runtime on other tissues to vary with dataset size and coverage complexity, including the number of aggregated samples. Peak memory ranged from approximately 0.75–12.8 GiB for SpliceCOV and 0.59–41.4 GiB for PELT. Segmentor3IsBack used approximately 0.03–2.36 GiB. GFPOP used approximately 272 MiB for bladder and 205 MiB for small intestine.

The SpliceCOV timings begin from precomputed junction BED and coverage BigWig files and include splice-site feature extraction, TSS/TES candidate generation and feature extraction, LightGBM inference, point-feature generation, and annotation-based evaluation.

## 4. Discussion

We introduced SpliceCOV, a supervised framework for filtering noisy RNA-Seq-derived transcript features to improve transcriptome assembly. Rather than predicting splice sites de novo, SpliceCOV evaluates candidate features already supported by RNA-Seq evidence and identifies which are most likely to represent true transcript features. For splice sites, candidate donor and acceptor sites are obtained directly from observed splice junctions, and a LightGBM classifier is used to distinguish well-supported splice sites from likely noisy junction-derived candidates. For TSS/TES, where no equivalent candidate set is provided by the junction input, SpliceCOV first uses a coverage-based heuristic to propose candidate boundaries and then applies a separate LightGBM classifier to filter them. This design reflects the main goal of SpliceCOV: not to replace existing segmentation or sequence-based splice-site prediction tools, but to remove transcriptional noise from RNA-Seq-derived features before transcriptome assembly.

This distinction is important because transcriptome assembly depends on the quality of the evidence supplied to the assembler. A permissive set of RNA-Seq features may preserve sensitivity, but it can also introduce spurious junctions and uncertain transcript boundaries into assembled transcript models. Conversely, overly aggressive filtering may remove genuine low-abundance or tissue-specific features. SpliceCOV is designed to improve this trade-off by retaining most annotated splice sites and transcript boundaries while reducing the number of likely false-positive features. When SpliceCOV-filtered features were used to guide StringTie, transcriptome assembly precision increased across GTEx tissues without compromising transcript recovery.

The use of LightGBM was motivated by both predictive performance and scalability. We tested alternative supervised classifiers, including logistic regression and random forest models, but LightGBM achieved stronger classification performance while remaining efficient on transcriptome-scale candidate sets. This advantage is important because SpliceCOV must score large numbers of candidate features across genome-wide RNA-Seq coverage and junction files. LightGBM also provides a practical balance among accuracy, runtime, memory use, and interpretability through feature importance analysis, making it well-suited for this RNA-Seq feature-filtering task.

SpliceCOV intentionally uses RNA-Seq-derived coverage and junction features rather than sequence features. This design choice follows from the purpose of the method. Sequence-based models, including deep learning approaches such as SpliceAI [36], SPLAM [37], and SegmentNT [38], are designed to evaluate splice-site potential from genomic sequence context. In contrast, SpliceCOV evaluates whether a candidate feature is supported in a particular RNA-Seq dataset or tissue context. A genomic sequence may contain a strong splice motif but show no evidence of usage in the RNA-Seq data being assembled. Conversely, a weak or noncanonical site may still be supported by reproducible junction and coverage evidence. Therefore, sequence features and SpliceCOV address related but distinct questions. Sequence information is complementary and may improve future versions of the model, but it was not included here because our goal was to test whether RNA-Seq evidence alone can effectively filter noisy candidate features for transcriptome assembly.

Although SpliceCOV improves agreement with existing annotations, annotation overlap is an imperfect measure of correctness. Some predictions that do not overlap CHESS or GENCODE may represent genuine but currently unannotated transcriptional features, particularly in tissues with deeper RNA-Seq coverage or in regions with complex isoform structure. For this reason, non-overlapping predictions should be interpreted as annotation-discordant or likely false-positive calls rather than definitive errors. Future work should incorporate orthogonal experimental evidence, including CAGE data [39] for transcription start sites and poly(A)-focused assays [40] for transcript end sites, to better evaluate transcript-boundary predictions.

Our study is based on short-read RNA-Seq and therefore does not directly evaluate the effect of read length. GTEx v8 was generated using a fixed short-read sequencing protocol. Because SpliceCOV relies on local coverage and junction patterns rather than sequence context, it may still recognize similar transcriptional features in long-read datasets if these local patterns are preserved. However, long-read sequencing introduces distinct error profiles and coverage characteristics that were not represented during training, so the current model may not identify artifacts that are specific to PacBio or Oxford Nanopore data. Longer reads may nevertheless provide stronger evidence for transcript structure because individual reads can span multiple splice junctions or entire transcript isoforms. Future work could retrain SpliceCOV on long-read datasets and incorporate long-read-specific features such as full-length molecule support and co-occurrence of multiple splice junctions within individual reads.

Several limitations of the current study should be noted. First, we did not directly benchmark SpliceCOV against sequence-based splice-site prediction methods such as SpliceAI and SPLAM. These methods use genomic sequence rather than RNA-Seq coverage and junction evidence and therefore address a complementary prediction problem, but direct comparison would help clarify the relative strengths of sequence-based and RNA-Seq-based filtering approaches. Second, the present study was evaluated primarily using short-read RNA-Seq and reference annotations and did not include independent validation using long-read transcript sequencing. Finally, TSS/TES predictions were not independently validated using orthogonal transcript-boundary resources such as CAGE or PolyASite. Incorporating these datasets in future work will be important for assessing transcript-boundary accuracy independently of reference annotations and for guiding further development of SpliceCOV.

The effect of SpliceCOV guidance on transcriptome assembly should be interpreted in the context of StringTie’s existing assembly procedure. SpliceCOV does not modify the underlying read alignments or StringTie’s core reconstruction algorithm; instead, it only changes the external transcript-feature evidence supplied through the --ptf option. It is therefore expected to refine assemblies rather than produce large changes in transcript recovery. Across human GTEx tissues, transcript-level precision increased by approximately 5–8 percentage points, whereas transcript-level sensitivity and the number of reference-matching transcripts remained nearly unchanged. Although this improvement may appear modest, it corresponds to the removal of thousands of false-positive transcript models in samples containing on the order of 20,000 reference-matching transcripts. Because these excess transcripts are often low-abundance and sample-specific, they can accumulate substantially when assemblies are merged across many samples, inflating tissue-level transcript catalogs with spurious isoforms. Thus, even modest per-sample gains in precision can meaningfully improve the quality of comprehensive transcriptome annotations.

## 5. Conclusions

SpliceCOV provides a supervised framework for filtering RNA-Seq-derived splice sites and transcript boundaries before transcriptome assembly. Rather than treating every observed junction or coverage change as reliable evidence, SpliceCOV uses coverage- and junction-derived features to distinguish annotation-supported transcript features from likely noisy candidates. Across GTEx tissues, the method retained most annotation-supported splice sites while substantially reducing non annotation-supported candidates, and its behavior remained consistent across tissues with different sample depths and on chromosomes excluded from model training.

When SpliceCOV-filtered features were supplied to StringTie, transcript-level precision increased across the evaluated human tissues while transcript sensitivity and the number of reference-matching transcripts remained largely unchanged. The human-trained model also transferred to mouse RNA-Seq without retraining, although the downstream assembly improvement was smaller than in human data. Together, these results show that supervised filtering of RNA-Seq-derived transcript features can reduce noisy evidence before assembly while preserving transcript recovery.

## Data Availability

The data used in this study are available through the repositories described below. Human GTEx v8 RNA sequencing data used in this study are available through controlled access via dbGaP/AnVIL under accession phs000424.v8.p2, and mouse RNA sequencing data were obtained from the ENCODE Project. Human reference transcript annotations were obtained from CHESS v3.1.3, and mouse reference annotations were obtained from GENCODE. SpliceCOV source code is freely available on GitHub (https://github.com/celinehohzm/SpliceCOV). Scripts used to reproduce the analyses, benchmarks, and figures presented in this study are also available in the Zenodo archive (https://zenodo.org/records/22738075) (accessed on 10 October 2026). The reproducibility archive contains the scripts and configuration needed to reproduce the analyses underlying Figure 3, Figure 4, Figure 5, Figure 6, Figure 7 and Figure 8, including benchmark evaluation, metric calculation, statistical testing, figure generation, and the chromosome-holdout comparison. Where configured, the workflow can also rerun prediction steps for SpliceCOV and the comparison methods from supplied junction and coverage inputs. SRR accession numbers and metadata for the GTEx samples used in this study are provided in the Supplementary Data.

## Supplementary Materials

The following supporting information can be downloaded at: https://www.mdpi.com/article/10.3390/genes17101307/s1, Supplementary File S1 contains Supplementary Figure S1–S9. Supplementary File S2 contains the SRR accession numbers and metadata for the GTEx samples used in this study. The file includes the following columns: SRR (unique SRA sequencing-run accession), body_site (anatomical site or cell preparation), histological_type (tissue category used for sample aggregation), LibraryLayout (paired-end or single-end sequencing), is_tumor (source-reported tumor designation), sex (source-reported sex), and BioSample (NCBI BioSample accession). Supplementary Figure S1. Kernel density estimates (KDEs) of key splice-site features for true-positive (matching splice sites, green) versus false-positive (non-matching splice sites, red) calls in three GTEx tissues (bladder, small intestine, thyroid). Columns correspond to tissues; rows correspond to features (all quantile-transformed). Supplementary Figure S2. Sensitivity–precision behavior of the comparison methods across method-specific stringency parameters. The analysis was performed on small-intestine chromosome 11. For each method, all other parameters were held fixed while the principal stringency parameter was varied. PELT penalty multipliers of 0.25–1024, GFPOP beta multipliers of 0.25–1024, Segmentor3IsBack target densities of 1–374 changepoints/Mb, and DeCAFS beta multipliers of 0.25–1024 were evaluated. The SpliceCOV operating point at its default probability threshold of 0.4 is shown for reference. Supplementary Figure S3. Mouse ENCODE RNA-Seq datasets used for the cross-species evaluation of SpliceCOV. The table lists the ENCODE experiment accession, spleen-derived cell population, mouse strain, sex, sequencing read length, library type, number of BAM files analyzed, and animal age. All datasets were generated from B6CASTF1/J mice using 101-bp single-end, reverse-stranded total RNA-seq. In total, 20 BAM files from seven ENCODE experiment accessions were included in the analysis. Supplementary Figure S4. Performance of splice-site identification on multi-sample aggregated RNA-Seq coverage in bladder and small intestine using MANE Select v1.5, RefSeq NM/NR, and the complete RefSeq annotation as alternative reference annotations. Splice-site filtering performance is shown for PELT, Segmentor3IsBack, GFPOP, and SpliceCOV. (A–D) Evaluation using MANE Select v1.5 as the reference annotation: (A) true-positive (TP) and false-positive (FP) counts for bladder, (B) corresponding sensitivity, precision, accuracy, and F1 score for bladder, (C) TP and FP counts for small intestine, and (D) corresponding performance metrics for small intestine. (E–H) Evaluation using RefSeq NM/NR as the reference annotation: (E,F) bladder and (G,H) small intestine, with count-based results shown in panels E and G and performance metrics shown in panels F and H. (I–L) Evaluation using the complete RefSeq annotation as the reference: (I,J) bladder and (K,L) small intestine, with count-based results shown in panels I and K and performance metrics shown in panels J and L. In the count panels, the baseline represents all splice sites inferred from RNA-Seq alignments before filtering. Colors and symbols indicate methods as shown in the legends. Supplementary Figure S5. Effect of RNA-Seq read depth on SpliceCOV splice-site filtering performance. Aligned reads from one bladder RNA-Seq sample (SRR1071717) and one small-intestine RNA-Seq sample (SRR1071181) were randomly downsampled to retain 10%, 25%, 50%, 75%, or 100% of the original aligned reads, and SpliceCOV was applied using the same trained model and decision threshold. (A) Numbers of true-positive and false-positive splice-site predictions in bladder across downsampling levels. (B) Corresponding sensitivity, precision, and F1 score for bladder. (C) Numbers of true-positive and false-positive splice-site predictions in small intestine across downsampling levels. (D) Corresponding sensitivity, precision, and F1 score for small intestine. Supplementary Figure S6. Comparison of SpliceCOV with changepoint-based methods and a simple junction-support threshold for splice-site filtering in bladder and small intestine. (A) Numbers of true-positive and false-positive splice-site predictions in bladder for PELT, Segmentor3IsBack, GFPOP, SpliceCOV, and a baseline filter retaining junctions supported by more than five reads. The gray bars indicate the unfiltered junction-derived baseline candidate set. (B) Corresponding sensitivity, precision, accuracy, and F1 score for bladder. (C) Numbers of true-positive and false-positive predictions in small intestine using the same methods and junction-support threshold. (D) Corresponding performance metrics for small intestine. Supplementary Figure S7. LightGBM gain-based feature importance for the SpliceCOV splice-site classifier. Feature importance was calculated from the total gain contributed by splits using each feature and normalized to sum to 100%. Gain-based importance reflects the relative contribution of features to tree-split improvement and should not be interpreted as a causal measure of biological importance. Supplementary Figure S8. TSS/TES detection performance using the full genome-wide set of 131,927 CHESS-annotated transcript start and end sites as the recall denominator. Sensitivity, precision, and F1 score are shown for PELT, Segmentor3IsBack, SpliceCOV, and DeCAFS in (A) bladder and (B) small intestine. Sensitivity was calculated as the number of CHESS-overlapping predicted TSS/TES divided by all 131,927 annotated CHESS TSS/TES positions, while precision was calculated as the fraction of predicted TSS/TES that overlapped CHESS annotations. Because the denominator includes annotated transcript boundaries from genes that may not be expressed in the evaluated tissue, the sensitivity values should be interpreted as conservative lower bounds rather than tissue-specific recall. Supplementary Figure S9. Performance benchmarks of SpliceCOV, PELT, and Segmentor3IsBack across GTEx tissues. (A) Run time (minutes) for each method on TieCov-aggregated junctions from 16 GTEx tissues (sample size in parentheses). (B) Peak memory usage (MB) measured during each run. All benchmarks were conducted on a server equipped with an Intel^®^ Xeon^®^ Gold 6248R CPU @ 3.00 GHz (24 cores, 48 threads), 1 TB RAM, running RHEL 8 (x86_64).

## Abbreviations

The following abbreviations are used in this manuscript:

BAM	Binary Alignment Map
BED	Browser Extensible Data
CAGE	Cap analysis of gene expression
CHESS	Comprehensive Human Expressed SequenceS
DeCAFS	Detecting Changes in Autocorrelated and Fluctuating Signals
DNA	Deoxyribonucleic acid
EFB	Exclusive Feature Bundling
ENCODE	Encyclopedia of DNA Elements
FP	False positive
FN	False negative
TN	True negative
GBDT	Gradient-boosted decision tree
GENCODE	GENCODE gene annotation project
GFPOP	Generalized Functional Pruning Optimal Partitioning
GOSS	Gradient-based One-Side Sampling
GTEx	Genotype-Tissue Expression
KDE	Kernel density estimation
LightGBM	Light Gradient Boosting Machine
PELT	Pruned Exact Linear Time
PTF	Point-feature
RNA	Ribonucleic acid
SRR	Sequence Read Archive run accession
SS	Splice site
TES	Transcription end site
TP	True positive
TSS	Transcription start site
