# Supplementary material for: SpliceCOV: Supervised Filtering of RNA-Seq-Derived Transcript Features Improves Transcriptome Assembly
Source: Genes (Basel). 2026 Oct 20;17(10):1307. doi: 10.3390/genes17101307 (PMC13704264; doi:10.3390/genes17101307)
Supplement: Supplementary file 1 [file genes-17-01307-s001.zip › SpliceCOV_Supplementary_File_1.pdf]

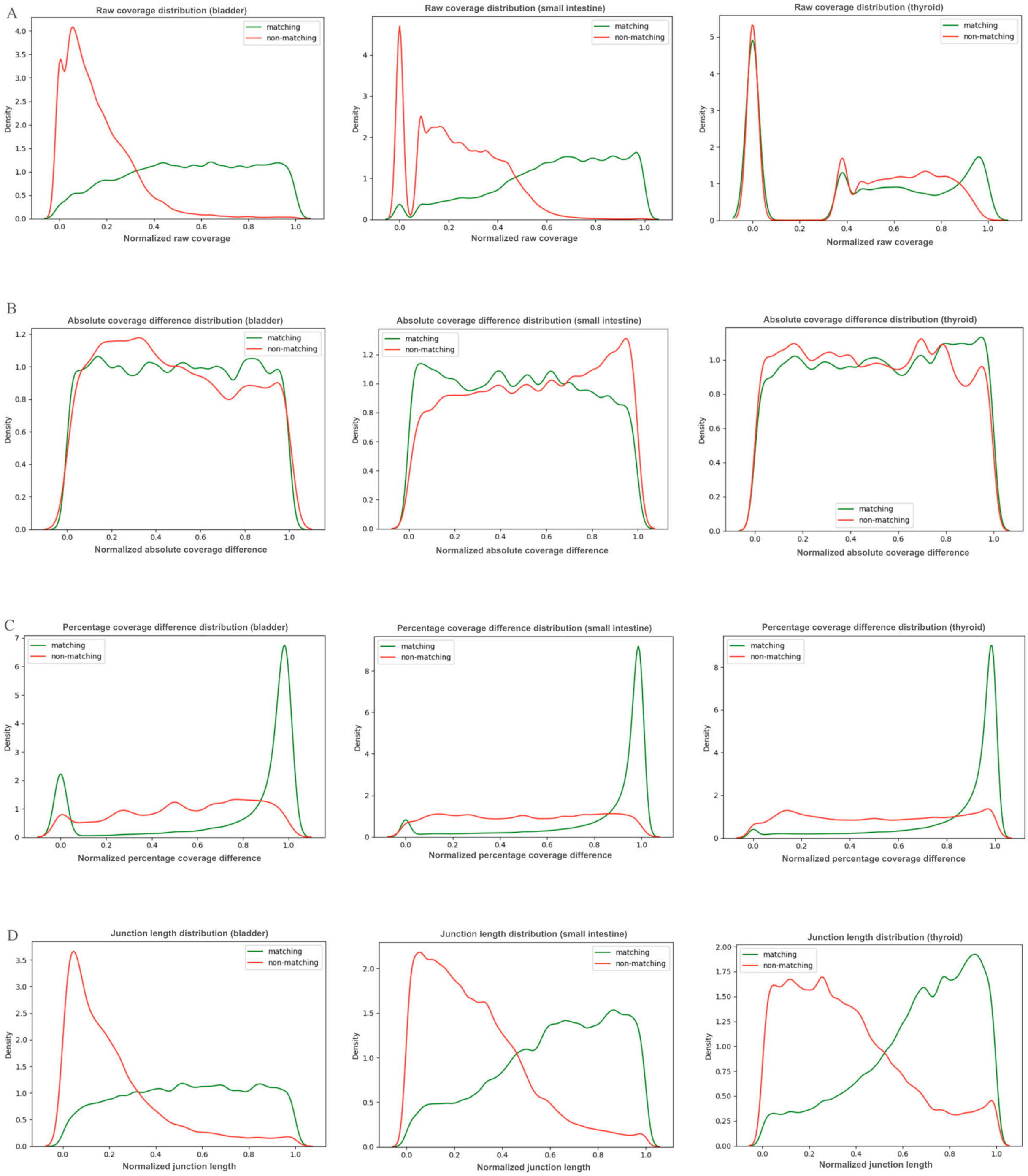
A
Raw coverage distribution (bladder)
Raw coverage distribution (small intestine)
Raw coverage distribution (thyroid)
matching
non-matching
Density
Normalized raw coverage
B
Absolute coverage difference distribution (bladder)
Absolute coverage difference distribution (small intestine)
Absolute coverage difference distribution (thyroid)
Normalized absolute coverage difference
C
Percentage coverage difference distribution (bladder)
Percentage coverage difference distribution (small intestine)
Percentage coverage difference distribution (thyroid)
Normalized percentage coverage difference
D
Junction length distribution (bladder)
Junction length distribution (small intestine)
Junction length distribution (thyroid)
Normalized junction length

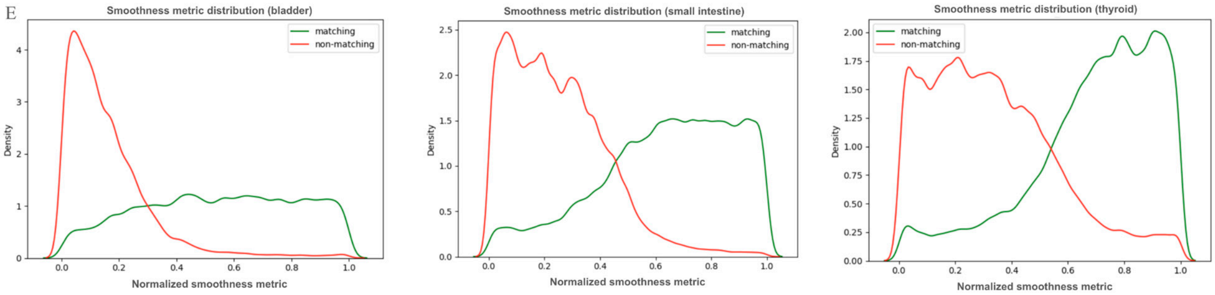

Supplementary Figure S1. Kernel density estimates (KDEs) of key splice-site features for true-positive (matching splice sites, green) versus false-positive (non-matching splice sites, red) calls in three GTEx tissues (bladder, small intestine, thyroid). Columns correspond to tissues; rows correspond to features (all quantile-transformed).

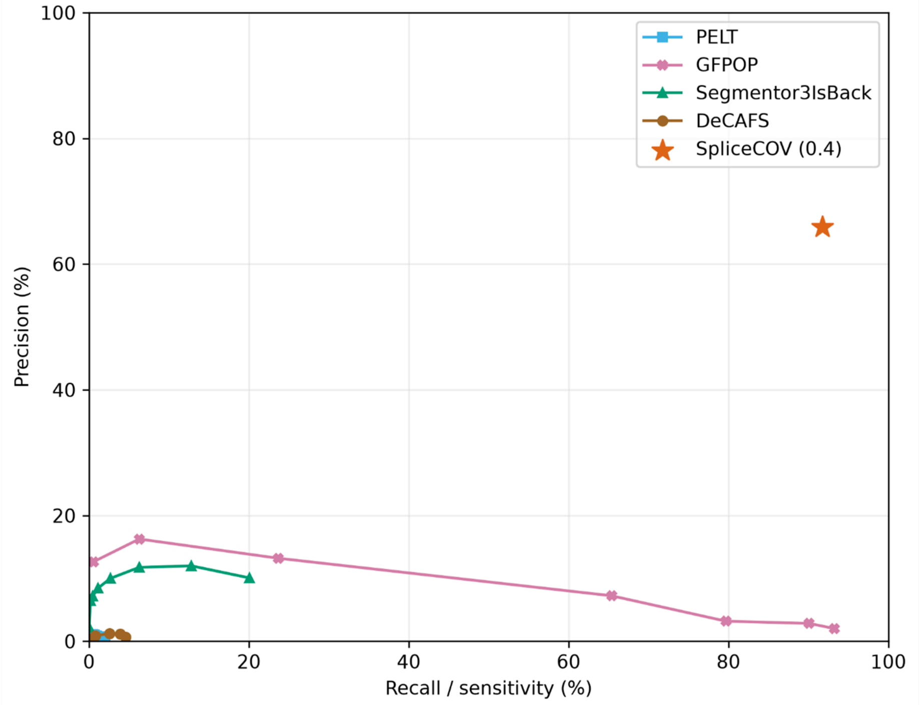

Supplementary Figure S2. Sensitivity–precision behavior of the comparison methods across method-specific stringency parameters. The analysis was performed on small-intestine chromosome 11. For each method, all other parameters were held fixed while the principal stringency parameter was varied. PELT penalty multipliers of 0.25–1024, GFPOP beta multipliers of 0.25–1024, Segmentor3IsBack target densities of 1–374 changepoints/Mb, and

DeCAFS beta multipliers of 0.25–1024 were evaluated. The SpliceCOV operating point at its default probability threshold of 0.4 is shown for reference.

ENCODE accession	Tissue / cell preparation	Strain	Sex	Read length (bp)	Library type	BAMs analysed	Age (days)
ENCSR703ACF	Spleen; $CD19^+$ B cells	B6CASTF1/J	Female	101	Total RNA-seq; single-end; reverse-stranded	3	55, 62
ENCSR788PEE	Spleen; $CD19^+$ B cells	B6CASTF1/J	Female	101	Total RNA-seq; single-end; reverse-stranded	3	67
ENCSR243UCH	Spleen; $CD19^+$ B cells	B6CASTF1/J	Male	101	Total RNA-seq; single-end; reverse-stranded	3	62
ENCSR092BBW	Spleen; $CD19^+$ B cells	B6CASTF1/J	Male	101	Total RNA-seq; single-end; reverse-stranded	4	67
ENCSR034MGF	Spleen; $CD3^+$ T cells	B6CASTF1/J	Female	101	Total RNA-seq; single-end; reverse-stranded	3	66
ENCSR377UPT	Spleen; $CD3^+$ T cells	B6CASTF1/J	Male	101	Total RNA-seq; single-end; reverse-stranded	3	66
ENCSR968ZOA	Spleen; naive resting $CD4^+$ $\alpha\beta$ T cells	B6CASTF1/J	Female	101	Total RNA-seq; single-end; reverse-stranded	1	57

Supplementary Figure S3. Mouse ENCODE RNA-Seq datasets used for the cross-species evaluation of SpliceCOV. The table lists the ENCODE experiment accession, spleen-derived cell population, mouse strain, sex, sequencing read length, library type, number of BAM files analyzed, and animal age. All datasets were generated from B6CASTF1/J mice using 101-bp single-end, reverse-stranded total RNA-seq. In total, 20 BAM files from seven ENCODE experiment accessions were included in the analysis.

MANE Select v1.5 as reference

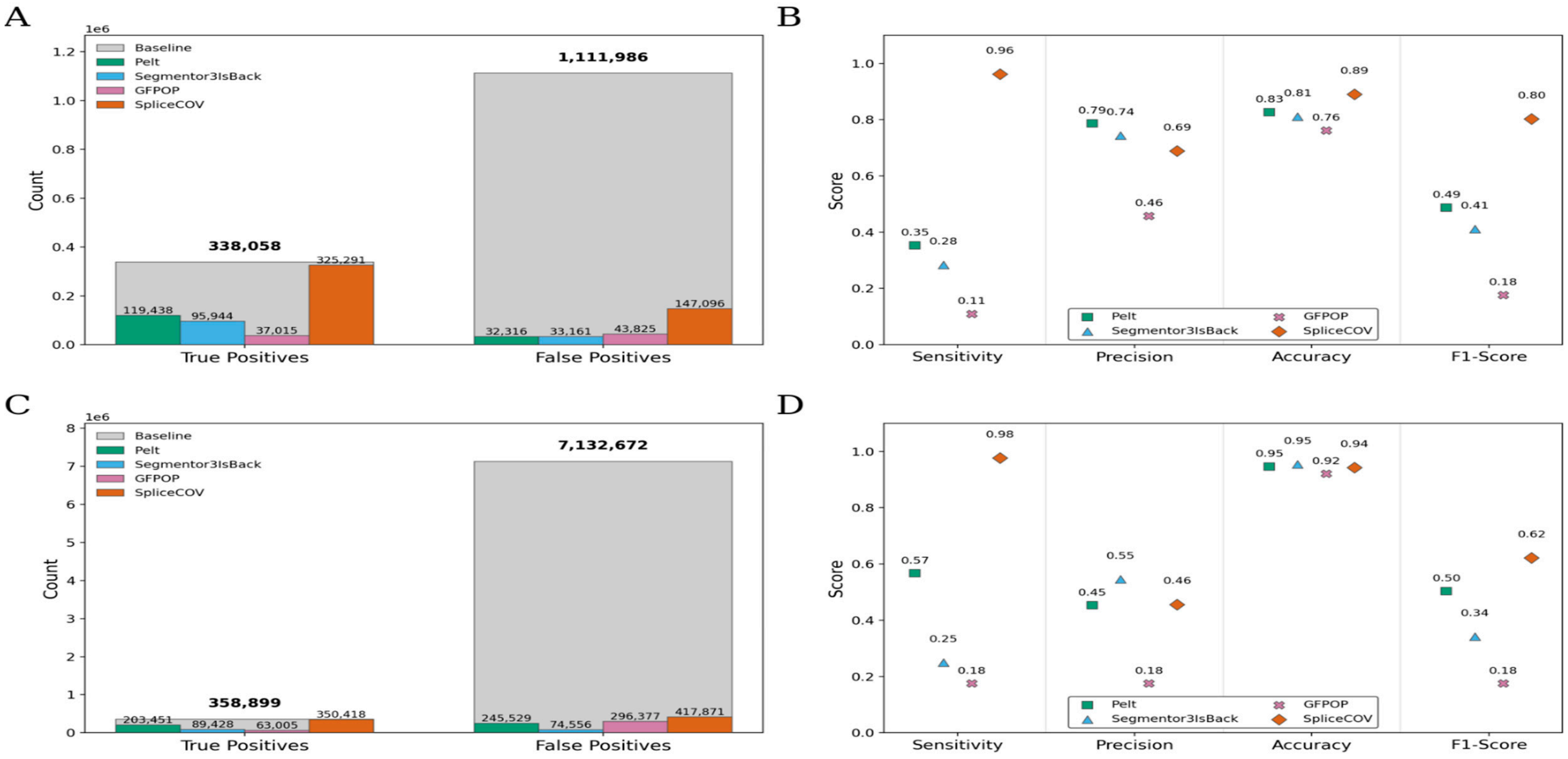

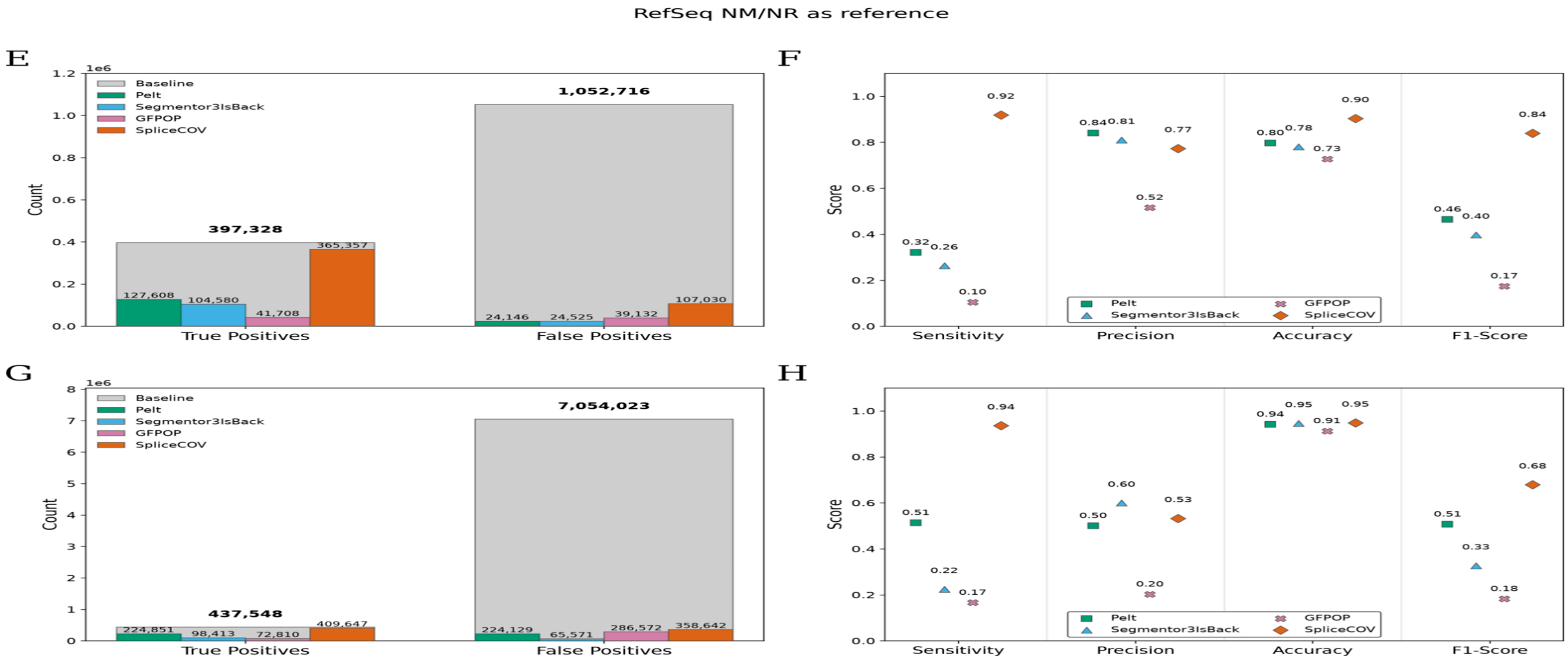

RefSeq NM/NR as reference
E
F
G
H
Baseline
Pelt
Segmentor3IsBack
GFPOP
SpliceCOV
Count
1e6
397,328
127,608
104,580
41,708
365,357
1,052,716
24,146
24,525
39,132
107,030
True Positives
False Positives
Score
Sensitivity
Precision
Accuracy
F1-Score
0.32
0.26
0.10
0.92
0.84
0.81
0.52
0.77
0.80
0.78
0.73
0.90
0.46
0.40
0.17
0.84
437,548
224,851
98,413
72,810
409,647
7,054,023
224,129
65,571
286,572
358,642
0.51
0.22
0.17
0.94
0.50
0.60
0.20
0.53
0.94
0.95
0.91
0.95
0.51
0.33
0.18
0.68

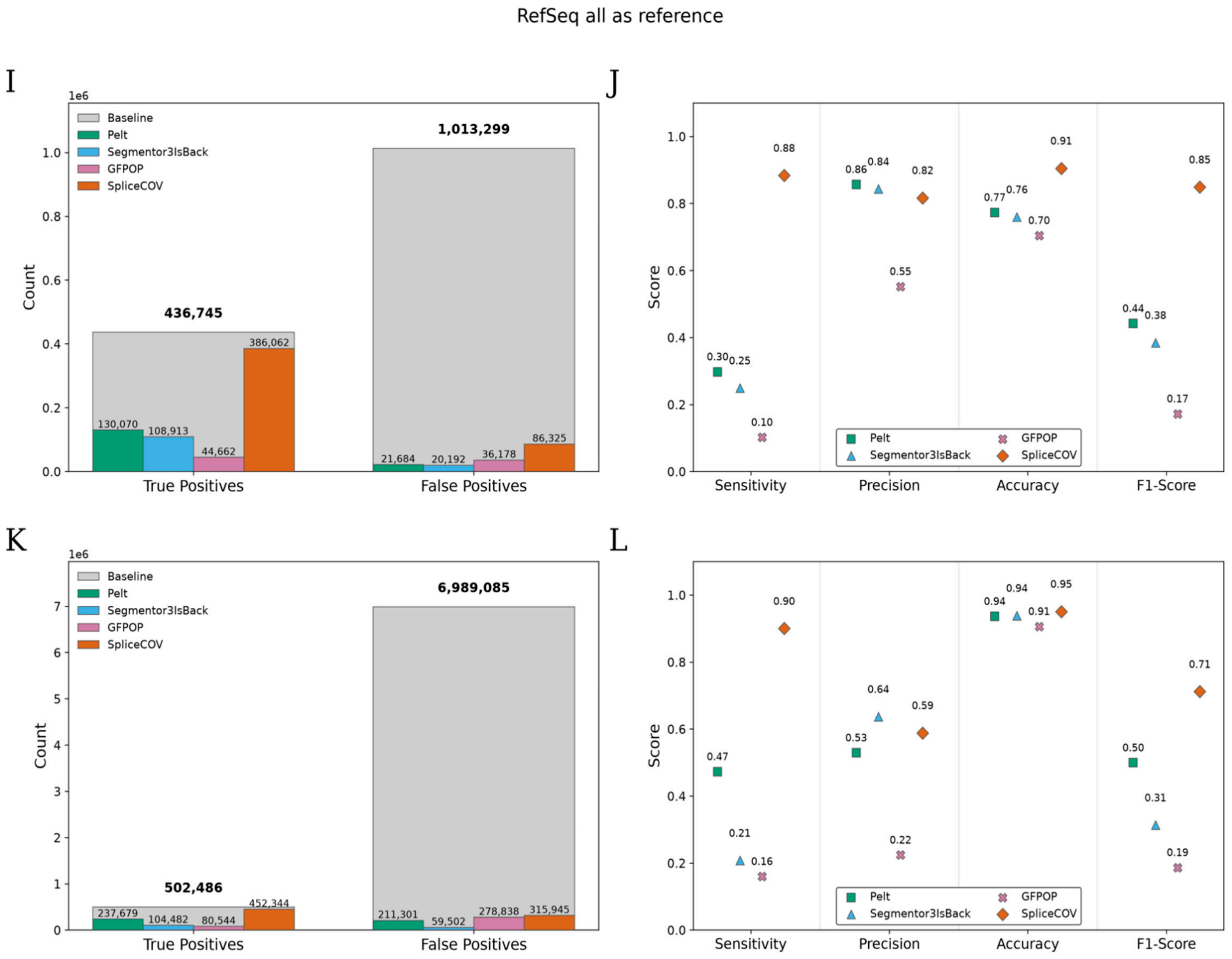

Supplementary Figure S4. Performance of splice-site identification on multi-sample aggregated RNA-Seq coverage in bladder and small intestine using MANE Select v1.5, RefSeq NM/NR, and the complete RefSeq annotation as alternative reference annotations. Splice-site filtering performance is shown for PELT, Segmentor3IsBack, GFPOP,

and SpliceCOV. (A–D) Evaluation using MANE Select v1.5 as the reference annotation: (A) true-positive (TP) and false-positive (FP) counts for bladder, (B) corresponding sensitivity, precision, accuracy, and F1 score for bladder, (C) TP and FP counts for small intestine, and (D) corresponding performance metrics for small intestine. (E–H) Evaluation using RefSeq NM/NR as the reference annotation: (E,F) bladder and (G,H) small intestine, with count-based results shown in panels E and G and performance metrics shown in panels F and H. (I–L) Evaluation using the complete RefSeq annotation as the reference: (I,J) bladder and (K,L) small intestine, with count-based results shown in panels I and K and performance metrics shown in panels J and L. In the count panels, the baseline represents all splice sites inferred from RNA-Seq alignments before filtering. Colors and symbols indicate methods as shown in the legends.

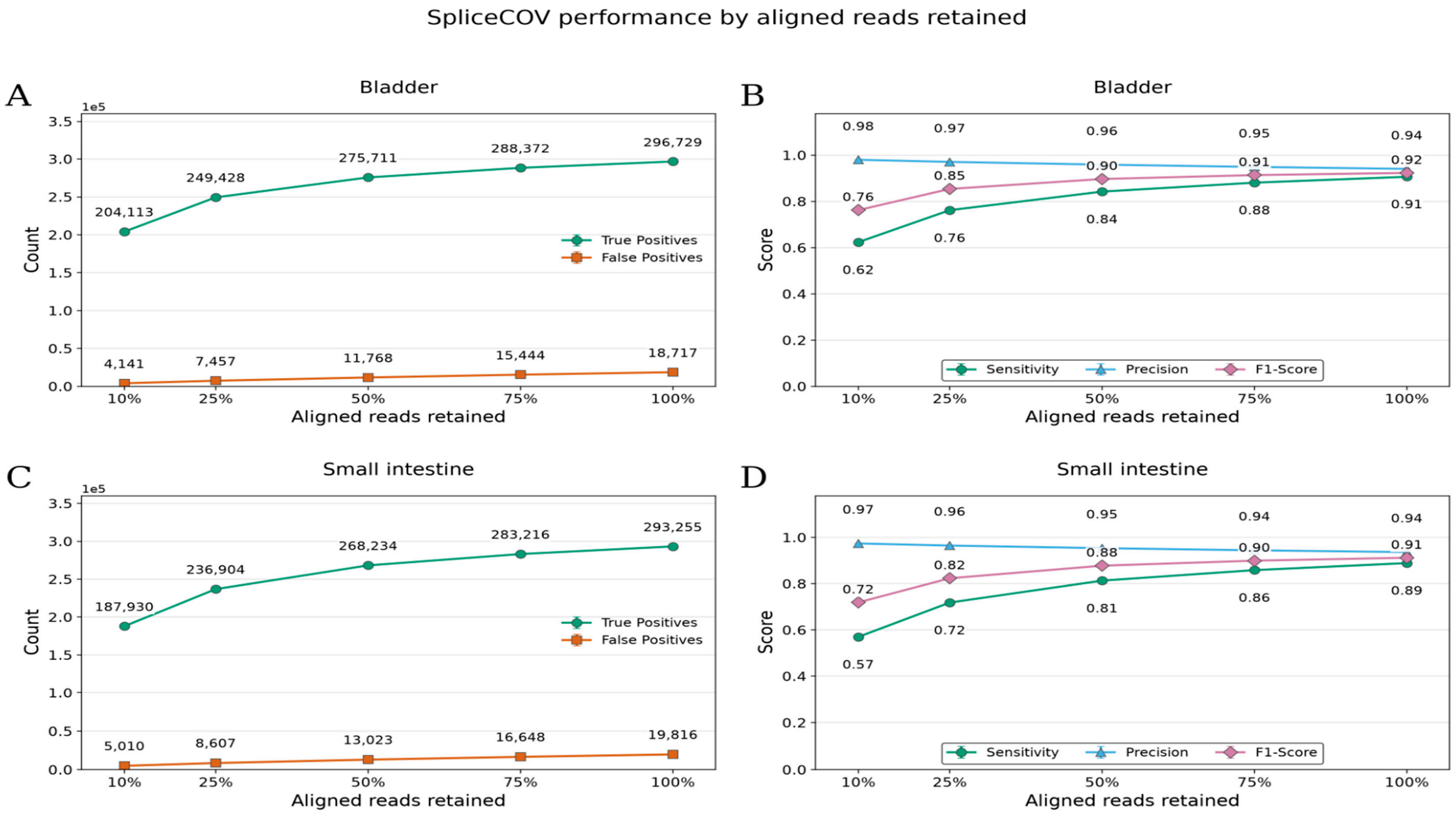
SpliceCOV performance by aligned reads retained
A
Bladder
1e5
Count
204,113
249,428
275,711
288,372
296,729
4,141
7,457
11,768
15,444
18,717
True Positives
False Positives
Aligned reads retained
B
Bladder
Score
0.98
0.97
0.96
0.95
0.94
0.76
0.85
0.90
0.91
0.92
0.62
0.76
0.84
0.88
0.91
Sensitivity
Precision
F1-Score
Aligned reads retained
C
Small intestine
1e5
Count
187,930
236,904
268,234
283,216
293,255
5,010
8,607
13,023
16,648
19,816
True Positives
False Positives
Aligned reads retained
D
Small intestine
Score
0.97
0.96
0.95
0.94
0.94
0.72
0.82
0.88
0.90
0.91
0.57
0.72
0.81
0.86
0.89
Sensitivity
Precision
F1-Score
Aligned reads retained
10%
25%
50%
75%
100%

Supplementary Figure S5. Effect of RNA-Seq read depth on SpliceCOV splice-site filtering performance. Aligned reads from one bladder RNA-Seq sample (SRR1071717) and one small-intestine RNA-Seq sample (SRR1071181) were randomly downsampled to retain 10%, 25%, 50%, 75%, or 100% of the original aligned reads, and SpliceCOV was applied using the same trained model and decision threshold. (A) Numbers of true-positive and false-positive splice-site predictions in bladder across downsampling levels. (B) Corresponding sensitivity, precision, and F1 score for bladder. (C) Numbers of true-positive and false-positive splice-site predictions in small intestine across downsampling levels. (D) Corresponding sensitivity, precision, and F1 score for small intestine.

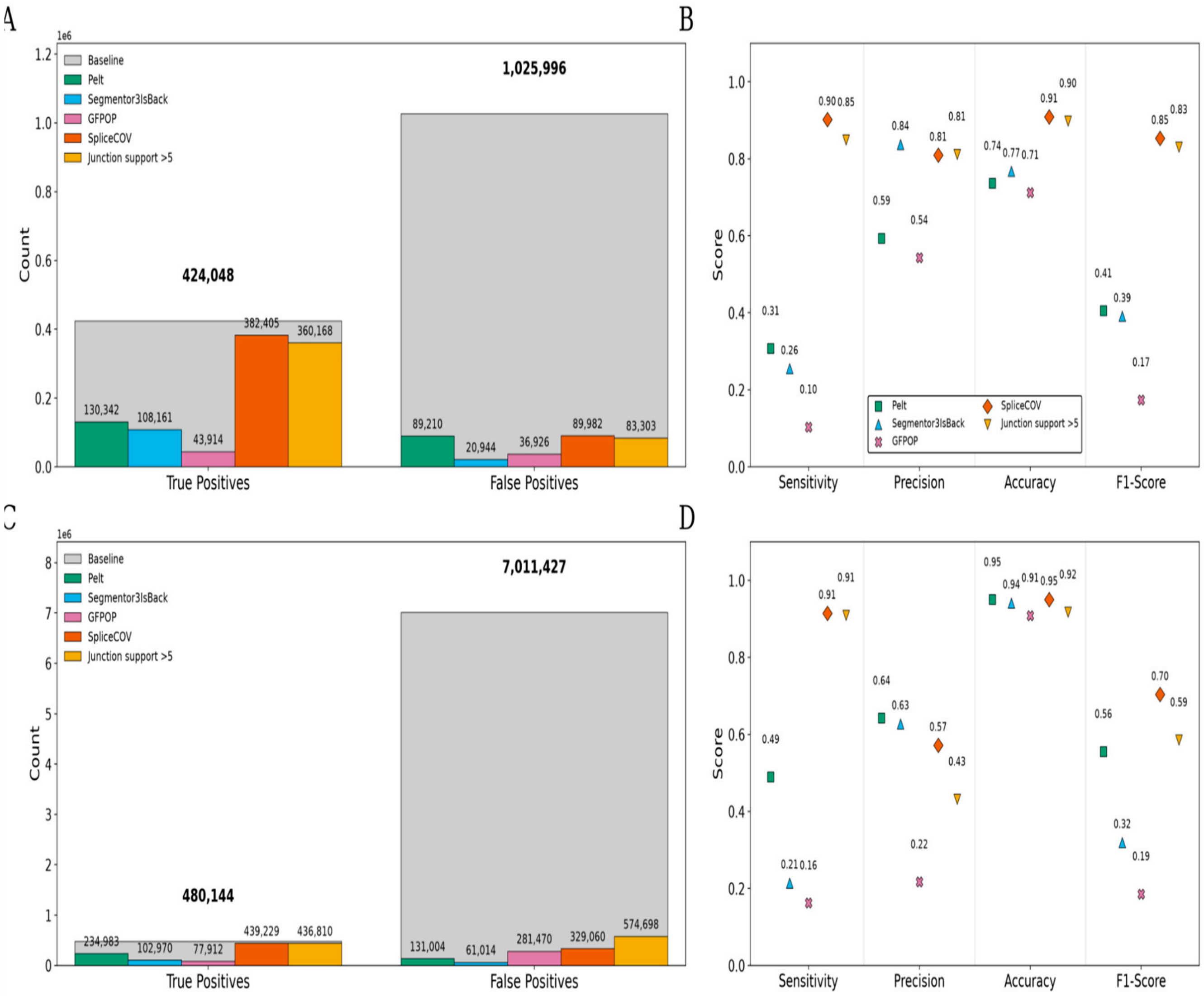
A
B
C
D
Baseline
Pelt
Segmentor3IsBack
GFPOP
SpliceCOV
Junction support >5
Count
Score
1e6
424,048
1,025,996
130,342
108,161
43,914
382,405
360,168
89,210
20,944
36,926
89,982
83,303
True Positives
False Positives
Sensitivity
Precision
Accuracy
F1-Score
480,144
7,011,427
234,983
102,970
77,912
439,229
436,810
131,004
61,014
281,470
329,060
574,698

Supplementary Figure S6. Comparison of SpliceCOV with changepoint-based methods and a simple junction-support threshold for splice-site filtering in bladder and small intestine. (A) Numbers of true-positive and false-positive splice-site predictions in bladder for PELT, Segmentor3IsBack, GFPOP, SpliceCOV, and a baseline filter retaining junctions supported by more than five reads. The gray bars indicate the unfiltered junction-derived baseline candidate set. (B) Corresponding sensitivity, precision, accuracy, and F1 score for bladder. (C) Numbers of true-positive and false-positive predictions in small intestine using the same methods and junction-support threshold. (D) Corresponding performance metrics for small intestine.

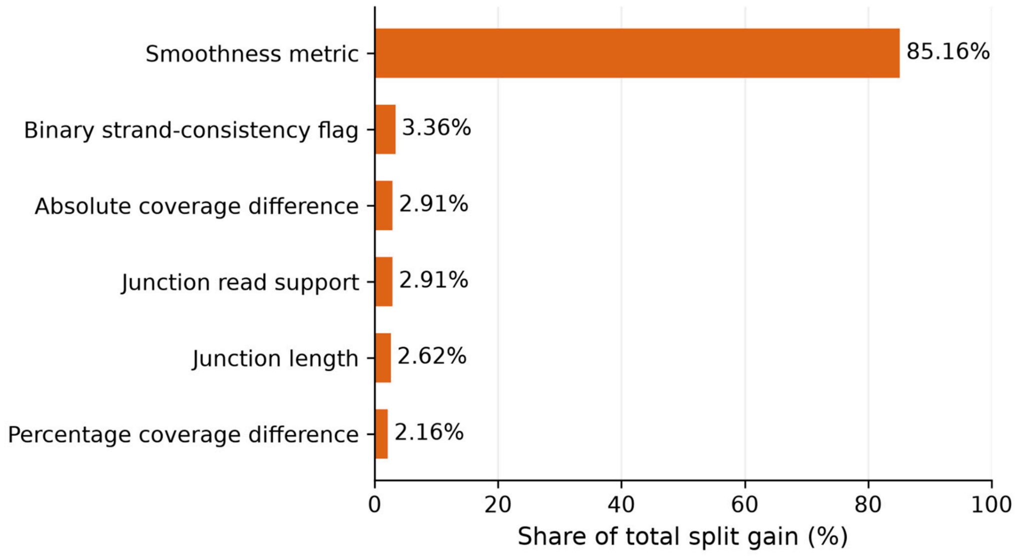

Supplementary Figure S7. LightGBM gain-based feature importance for the SpliceCOV splice-site classifier. Feature importance was calculated from the total gain contributed by splits using each feature and normalized to sum to 100%. Gain-based importance reflects the relative contribution of features to tree-split improvement and should not be interpreted as a causal measure of biological importance.

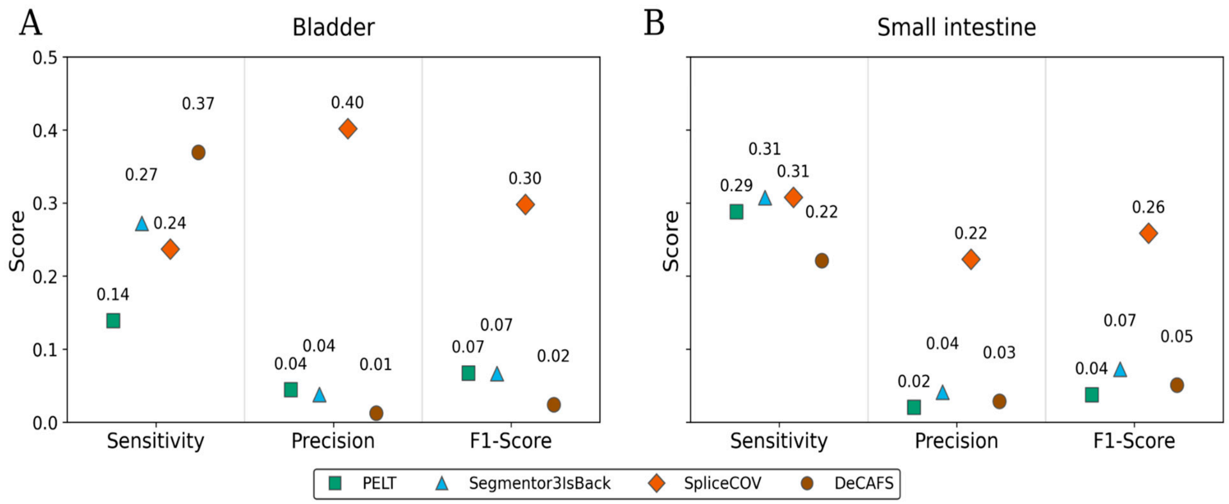

Supplementary Figure S8. TSS/TES detection performance using the full genome-wide set of 131,927 CHESS-annotated transcript start and end sites as the recall denominator. Sensitivity, precision, and F1 score are shown for PELT, Segmentor3IsBack, SpliceCOV, and DeCAFS in (A) bladder and (B) small intestine. Sensitivity was calculated as the number of CHESS-overlapping predicted TSS/TES divided by all 131,927 annotated CHESS TSS/TES positions, while precision was calculated as the fraction of predicted TSS/TES that overlapped CHESS annotations. Because the denominator includes annotated transcript boundaries from genes that may not be expressed in the evaluated tissue, the sensitivity values should be interpreted as conservative lower bounds rather than tissue-specific recall.

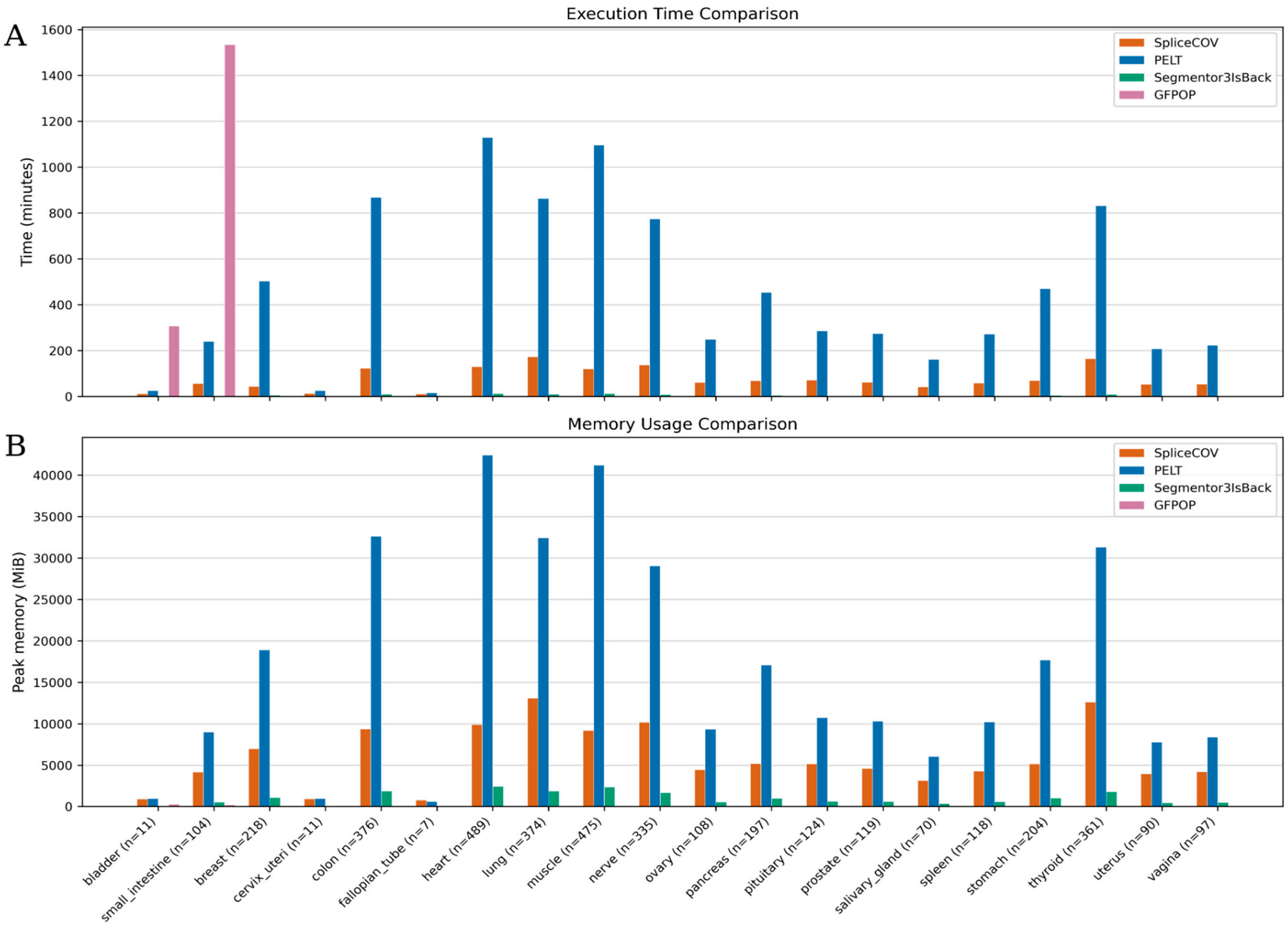
A
Execution Time Comparison
Time (minutes)
B
Memory Usage Comparison
Peak memory (MiB)
SpliceCOV
PELT
Segmentor3IsBack
GFPOP
bladder (n=11)
small_intestine (n=104)
breast (n=218)
cervix_uteri (n=11)
colon (n=376)
fallopian_tube (n=7)
heart (n=489)
lung (n=374)
muscle (n=475)
nerve (n=335)
ovary (n=108)
pancreas (n=197)
pituitary (n=124)
prostate (n=119)
salivary_gland (n=70)
spleen (n=118)
stomach (n=204)
thyroid (n=361)
uterus (n=90)
vagina (n=97)

Supplementary Figure S9. Performance benchmarks of SpliceCOV, PELT, and Segmentor3IsBack across GTEx tissues.
(A) Run time (minutes) for each method on TieCov-aggregated junctions from 16 GTEx tissues (sample size in parentheses).
(B) Peak memory usage (MB) measured during each run.
All benchmarks were conducted on a server equipped with an Intel® Xeon® Gold 6248R CPU @ 3.00 GHz (24 cores, 48 threads), 1 TB RAM, running RHEL 8 (x86_64).
